# Transcriptional mechanisms associated with seed dormancy and dormancy loss in the gibberellin-insensitive *sly1-2* mutant of *Arabidopsis thaliana*

**DOI:** 10.1371/journal.pone.0179143

**Published:** 2017-06-19

**Authors:** Sven K. Nelson, Camille M. Steber

**Affiliations:** 1 Molecular Plant Sciences Program, Washington State University, Pullman, Washington, United States of America; 2 USDA-ARS, Wheat Health, Genetics, and Quality Research Unit, Pullman, Washington, United States of America; 3 Department of Crop and Soil Science, Washington State University, Pullman, Washington, United States of America; Universidad Nacional Autonoma de Mexico, MEXICO

## Abstract

While widespread transcriptome changes were previously observed with seed dormancy loss, this study specifically characterized transcriptional changes associated with the increased seed dormancy and dormancy loss of the gibberellin (GA) hormone-insensitive *sleepy1-2* (*sly1-2*) mutant. The *SLY1* gene encodes the F-box subunit of an SCF E3 ubiquitin ligase needed for GA-triggered proteolysis of DELLA repressors of seed germination. DELLA overaccumulation in *sly1-2* seeds leads to increased dormancy that can be rescued without DELLA protein destruction either by overexpression of the GA receptor, *GA-INSENSITIVE DWARF1b* (*GID1b-OE*) (74% germination) or by extended dry after-ripening (11 months, 51% germination). After-ripening of *sly1* resulted in different transcriptional changes in early versus late Phase II of germination that were consistent with the processes known to occur. Approximately half of the transcriptome changes with after-ripening appear to depend on *SLY1*-triggered DELLA proteolysis. Given that many of these *SLY1*/GA-dependent changes are genes involved in protein translation, it appears that GA signaling increases germination capacity in part by activating translation. While *sly1-2* after-ripening was associated with transcript-level changes in 4594 genes over two imbibition timepoints, rescue of *sly1-2* germination by *GID1b-OE* was associated with changes in only 23 genes. Thus, a big change in *sly1-2* germination phenotype can occur with relatively little change in the global pattern of gene expression during the process of germination. Most *GID1b-OE*-responsive transcripts showed similar changes with after-ripening in early Phase II of imbibition, but opposite changes with after-ripening by late Phase II. This suggests that *GID1b-OE* stimulates germination early in imbibition, but may later trigger negative feedback regulation.

## Introduction

The evolution of seeds that carry plant embryos in a state of arrested growth was critical to the success of land plants and to agriculture [1]. Because seeds carry nutrient reserves for the germinating embryo, they are a convenient food source, comprising 70% of the human diet (reviewed in [2]). Seeds of temperate species can be dormant at maturity, meaning that they are unable to germinate under favorable conditions [3]. Dormancy prevents germination out of season (fall versus spring), and allows species to survive natural disasters as seeds in the soil. Seed dormancy can be lost through a period of dry storage called after-ripening, through a period of moist chilling called cold stratification, or through seed coat scarification. Seed dormancy is associated with high levels of the hormone abscisic acid (ABA) and germination is associated with high levels of the hormone gibberellin (GA) (reviewed in [4]). Increased ABA signaling or decreased GA signaling may promote seed dormancy through transcriptional regulation. Previous studies identified transcripts differentially regulated with dormancy loss through after-ripening [5–10]. This study examined changes in the global pattern of gene expression associated with seed dormancy and dormancy loss in a GA-insensitive mutant that over-accumulates DELLA protein repressors of seed germination. The events that occur during seed germination and GA signaling are reviewed to provide background for the transcriptome analysis.

Seed germination is both an event and a three-phase process (reviewed in [2]). Phase I involves the rapid uptake of water and cellular rehydration, and occurs in both dormant and non-dormant seeds. Phase II processes prepare the seed for germination, including roughly in order: the repair of DNA damage sustained during dehydration and storage, mitochondrial repair and initiation of cellular respiration, the mobilization of stored nutrient reserves, the initiation of transcription and protein translation, and lastly DNA synthesis and cell expansion in the radicle or embryonic root. Phase III begins with germination *per se*, embryo emergence from the seed, and includes the post-germinative processes of cell division, seedling growth, and the majority of nutrient mobilization. Dormant seeds undergo the Phase II processes of cellular restoration, DNA repair, initiation of respiration, and some RNA transcription and protein translation, but never reach Phase III. Differences in dormant seed Phase II processes likely prevent germination *per se*. Since dormant seeds will not complete the germination process, we will refer to ungerminated seeds undergoing Phase I and II as “imbibing seeds.” While imbibing in the cold breaks dormancy, *Arabidopsis thaliana* seeds do not reach Phase III during this cold stratification and eventually enter secondary dormancy [5,11]. Brief cold stratification (i.e. 3–5 d at 4°C) synchronizes Arabidopsis seeds in early Phase II [12,13]. GA stimulates multiple germination processes, including radicle cell elongation, cotyledon expansion, and the production of enzymes that mobilize stored reserves and weaken barrier tissues such as the aleurone and testa (reviewed in [14], [12,15]).

GA stimulates GA responses by negatively regulating DELLA (Asp-Glu-Leu-Leu-Ala) domain repressors of GA signaling (reviewed in [16]). In the GA biosynthesis mutant, *ga1-3*, DELLA protein levels are high and block GA responses including stem elongation, cell division, the transition to flowering, and seed germination [17,18]. GA treatment of *ga1-3* results in rapid DELLA proteolysis followed by rescue of phenotypes including germination [19–21]. The partially overlapping functions of the five Arabidopsis DELLA genes were defined based on differences in the ability of DELLA knockouts to rescue *ga1-3* phenotypes (reviewed in [22]). Only the DELLA *RGL2* (*RGA-LIKE2*) knockout rescued *ga1-3* germination in the light, but mutations in DELLAs *RGA* (*REPRESSOR OF GA1-3*) and *GAI* (*GA INSENSITIVE*) were also needed to rescue *ga1-3* dark germination [23].

DELLAs positively and negatively regulate transcription by interaction with other transcription factors involved in a wide range of processes [24–27]. While DELLA does not directly bind DNA, DELLA RGA chromatin immunoprecipitation (ChIP)-seq and ChIP-qPCR analysis revealed association with a wide range of promoter elements including cytokinin-regulated, GA-responsive, light-regulated, stress-regulated, and ABA-related genes [24,28]. Examples of DELLA-interactors include: 1) the basic helix-loop-helix (bHLH) transcription factors that regulate cell elongation in response to light, PIF3 (PHYTOCHROME INTERACTING FACTOR3), PIF4, PIL2 (PIF3-LIKE2), and PIF1/PIL5, 2) the regulator of brassinosteroid signaling BZR1 (BRASSINOZALE-RESISTANT1), 3) the negative regulator of jasmonic acid signaling JAZ1 (JASMONATE ZIM DOMAIN1), and 4) the MYB transcription factor GL1 (GLABRA1) and bHLH GL3 involved in trichome initiation (reviewed in [22], [25,29]).

GA triggers DELLA proteolysis via the ubiquitin-proteasome pathway. The GA receptor GID1 (GA-INSENSITIVE DWARF1) undergoes a conformational change upon GA binding, stimulating interaction with DELLA proteins [30–33]. The formation of the GID1-GA-DELLA complex causes DELLA recognition by the SLEEPY1 (SLY1) F-box subunit of an SCF (Skp, Cullin, F-box) E3 ubiquitin ligase [34–38]. The SCF^SLY1^ complex catalyzes DELLA polyubiquitination, targeting it for degradation via the 26S proteasome. GA-stimulated DELLA destruction lifts DELLA repression of GA responses. Like *ga1-3*, the triple knockout of the three Arabidopsis *GID1* receptors, *GID1a*, *GID1b*, and *GID1c*, causes failure to germinate unless the seed coat is cut [21,35,39,40]. The GA-insensitive *sly1* F-box mutants exhibit varying degrees of increased seed dormancy associated with failure to degrade DELLA protein. This study makes use of the *sly1-2* allele, a 2-bp deletion resulting in a frameshift and the loss of the last 40 of 151 amino acids, including the proposed DELLA binding site.

DELLA repression in the Arabidopsis *sly1* and the rice *gid2* F-box mutants can be lifted by non-proteolytic mechanisms [32,41,42]. Although *sly1* mutants accumulate more DELLA protein than *ga1-3* or *gid1a gid1b gid1c* triple mutants, they show weaker GA-insensitive phenotypes. This suggests that some GA signaling occurs in *sly1* mutants despite high DELLA protein levels [17,35,39,43]. The germination of *sly1-2* seeds can be rescued by two mechanisms that do not reduce DELLA protein accumulation, after-ripening and *GID1* gene overexpression [21,42]. One to two years of dry after-ripening are needed to relieve *sly1-2* dormancy, compared to two weeks of after-ripening in Landsberg *erecta* wild-type (L*er* wt). The *sly1-2* mutant allows us to look at after-ripening mechanisms that can function without DELLA destruction. Partial rescue of *sly1* germination by *GID1a*, *GID1b*, and *GID1c* overexpression (*GID1*-*OE*) was associated with increased GID1-GA-DELLA complex formation, suggesting that GID1 can inactivate DELLA repressors without SLY1-directed DELLA destruction. *GID1b-OE* rescued *sly1-2* seed germination better than *GID1a-OE* and *GID1c-OE*, likely because GID1b has higher affinity for GA and DELLA [42,44,45]. The model is that *sly1-2* after-ripening leads to increased GA hormone levels and higher GID1b protein levels, thereby inactivating DELLA via GID1-GA-DELLA complex formation [42,46]. If both after-ripening and *GID1b-OE* rescue germination through increased GID1-GA-DELLA complex formation, then they should result in overlapping changes in the global pattern of transcript abundances. Moreover, genes showing differential regulation with after-ripening and *GID1b-OE* in *sly1-2* may represent the non-proteolytic mechanisms needed to overcome DELLA repression of seed germination.

It is important to define specific pathways governing seed dormancy. This has been difficult given the plethora of expression changes observed in multiple Arabidopsis ecotypes, times in imbibition, and under multiple environmental conditions [5–8,47,48]. This problem is due to the fact that multiple genetic mechanisms cause dormancy in different species, genotypes, and at different times in the germination process. By identifying global patterns of transcript abundance associated with *sly1-2* dormancy and dormancy loss, this study teased apart DELLA-dependent dormancy mechanisms, as well as dormancy-release mechanisms that do not require DELLA destruction. Distinct transcriptional changes were observed during early and late Phase II of germination. Interestingly, *sly1-2* dormancy loss due to *GID1b*-overexpression was associated with changes in a very small set of transcripts. Thus, rescue of *sly1-2* germination can occur with little change in the transcriptome. Finally, gene ontology analysis revealed that GA and *SLY1* are important for increasing abundance of transcripts involved in protein translation with dormancy loss.

## Results

### Strategy for identifying SLY1-dependent and -independent regulation of seed dormancy

**Fig 1:**
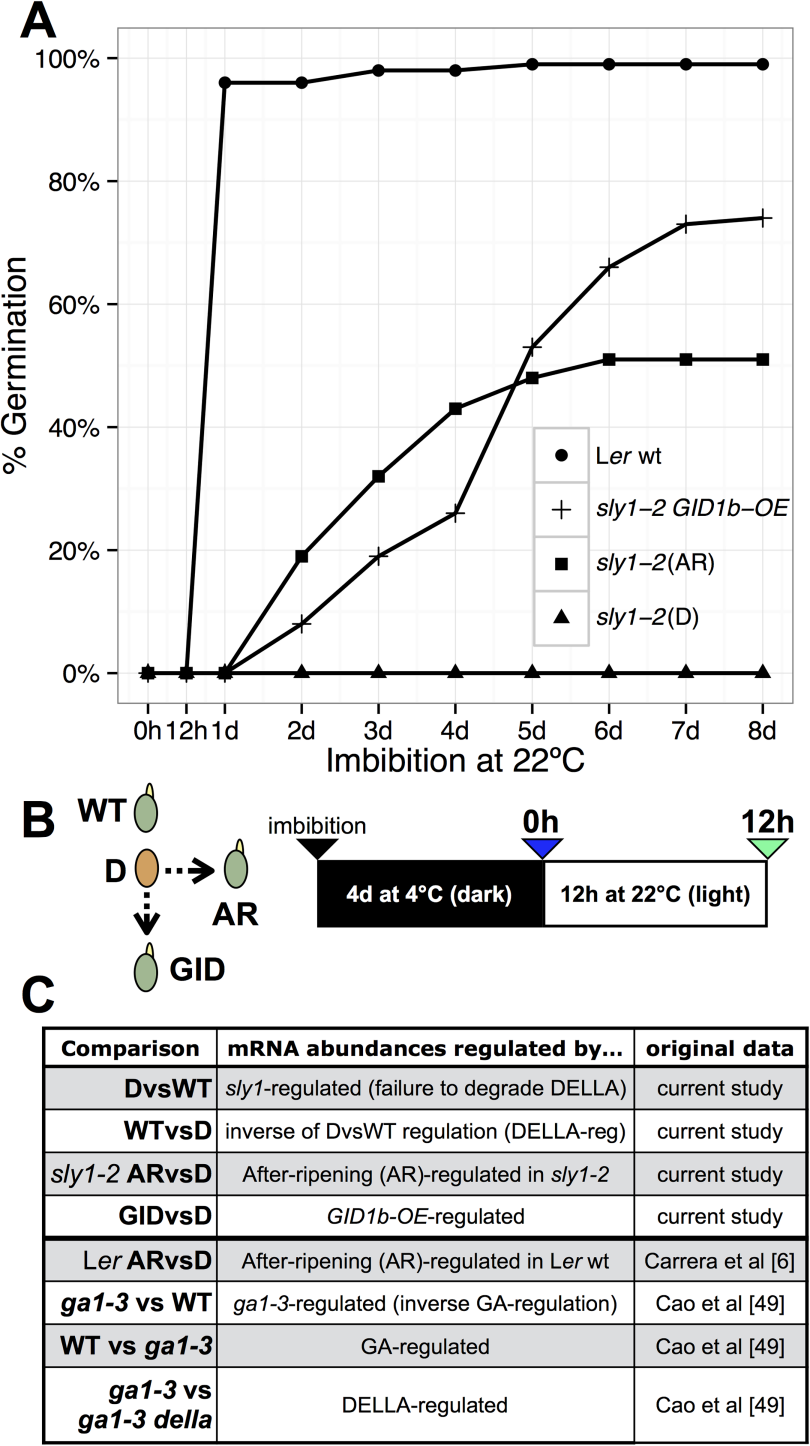
Experimental design for microarray analysis. (A) Germination of seeds used for microarray analysis including: 2-week-old L*er* wt, 2-week-old *sly1-2* (dormant), 19-month-old *sly1-2* (after-ripened), and 2-week-old *sly1-2 GID1b-OE* seeds. (B) For comparisons, the four seed samples are referred to as WT, D, AR, and GID, respectively. Seeds of each line were cold stratified for 4 days at 4°C in the dark and then transferred to the light at 22°C. Seeds were harvested for RNA extraction at the “0h” (blue) timepoint immediately after the cold stratification, and at the “12h” (green) timepoint (cold stratification followed by 12 h in the light at 22°C). (C) Experimental comparisons made in this paper, including comparisons from reanalysis of data from Carrera et al. [6] and Cao et al. [49].

Germination kinetics following cold stratification for 4 days at 4°C were determined for seeds of: a) non-dormant wild-type L*er* (WT) after-ripened for 2 weeks, b) dormant *sly1-2* after-ripened for 2 weeks (*sly1-2*(D)), c) non-dormant *sly1-2* after-ripened for 19 months (*sly1-2*(AR)), and d) non-dormant *sly1-2 GID1b-overexpressed* (*sly1-2 GID1b-OE*) after-ripened for 2 weeks (Fig 1A). WT showed neither testa rupture nor radicle emergence at 12h of imbibition, but achieved 96% germination within 24 h, indicating lack of seed dormancy. In contrast, the *sly1-2*(D) sample showed no germination after 8 d of imbibition, indicating failure to lose dormancy with the same 2 weeks (wk) of after-ripening. Germination of *sly1-2* was rescued to 74% by day 8 in *sly1-2 GID1b-OE* seeds and to 51% germination by day 6 by *sly1-2*(AR). Cold stratification was used to improve the synchrony of germination in *sly1-2 GID1b-OE*, *sly1-2*(AR), and L*er* wt. It should be noted that cold stratification does not rescue the germination of dormant *sly1-2* seeds.

Using these four sets of seeds, an experiment was designed to examine transcriptional mechanisms of seed dormancy and dormancy loss in the *sly1-2* mutant. Seeds were sampled at two imbibition timepoints representing early and late Phase II, and subjected to Affymetrix oligonucleotide-based microarray analysis. Cold stratification for 4 d at 4°C in the dark was used to synchronize seeds in Phase II [2]. Seeds at the “0 hour” (0h, early Phase II) timepoint were harvested immediately after cold stratification, while seeds at the “12 hour” (12h, late Phase II) timepoint were imbibed for 12 h in the light at 22°C after cold stratification (Fig 1B). These timepoints allowed examination of transcriptome differences during the early and late germination process, before germination *per se*.

This experimental design allowed comparisons of dormant and less dormant samples within the current study, and between this study and reanalyzed microarray datasets from previous studies (Fig 1C). D refers to *sly1-2*(D or Dormant) and AR refers to *sly1-2*(AR or After-ripened) for all comparisons but for the L*er* ARvsD from Carrera et al. [6]. WT refers to L*er* wt, while GID refers to *sly1-2 GID1b-OE*. Our 0h imbibition timepoint matched the seed imbibition conditions used by Cao et al. [49] for WT, *ga1-3*, and *ga1-3 della* transcriptomes (where *ga1-3 della* refers to *ga1-3 gai-t6 rga-t2 rgl1-1 rgl2-1*). The L*er* D and AR seeds used in Carrera et al. [6] were sampled at 24 h of imbibition without cold stratification representing late Phase II, allowing comparison with the *sly1-2* 12h timepoint as, in both cases, germination *per se* of non-dormant seeds occurred approximately 12 h after sampling.

### Transcriptome differences associated with *sly1-2* dormancy and after-ripening

The DvsWT comparison revealed transcriptome differences associated with the *sly1-2* dormancy phenotype, including 1741 up- and 1818 down-regulated transcripts at the 0h and 3247 up- and 3434 down-regulated transcripts at the 12h imbibition timepoint (S1 Table). Since the *sly1-2* mutant over-accumulates DELLA protein, these differentially-regulated genes may be indirectly considered DELLA-regulated genes. DELLA protein over-accumulation occurs in both *sly1-2* and *ga1-3* mutants, but only *sly1-2* germination can be rescued through after-ripening, likely via DELLA-proteolysis-independent GA signaling [21]. To compare the effects of DELLA accumulation in *sly1* to DELLA accumulation in *ga1-3*, we compared 0h DvsWT to *ga1-3* vs WT (S1A Fig). If the *ga1-3* and *sly1-2* germination phenotypes result solely from accumulation of DELLA repressors of seed germination, then these two mutations should result in nearly identical changes in transcript accumulation. Instead, the *sly1* DvsWT and *ga1-3* vs WT comparisons had a large but incomplete overlap of about 39% of either individual comparison. 60% of the DvsWT transcriptome changes were unique to the *sly1-2* comparison, while about 61% of the *ga1-3* vs WT changes were unique to the *ga1-3* comparison. Transcriptome differences between *ga1-3* and *sly1-2* may shed light on the role of DELLA-proteolysis-independent GA signaling in *sly1* dormancy loss.

**Fig 2:**
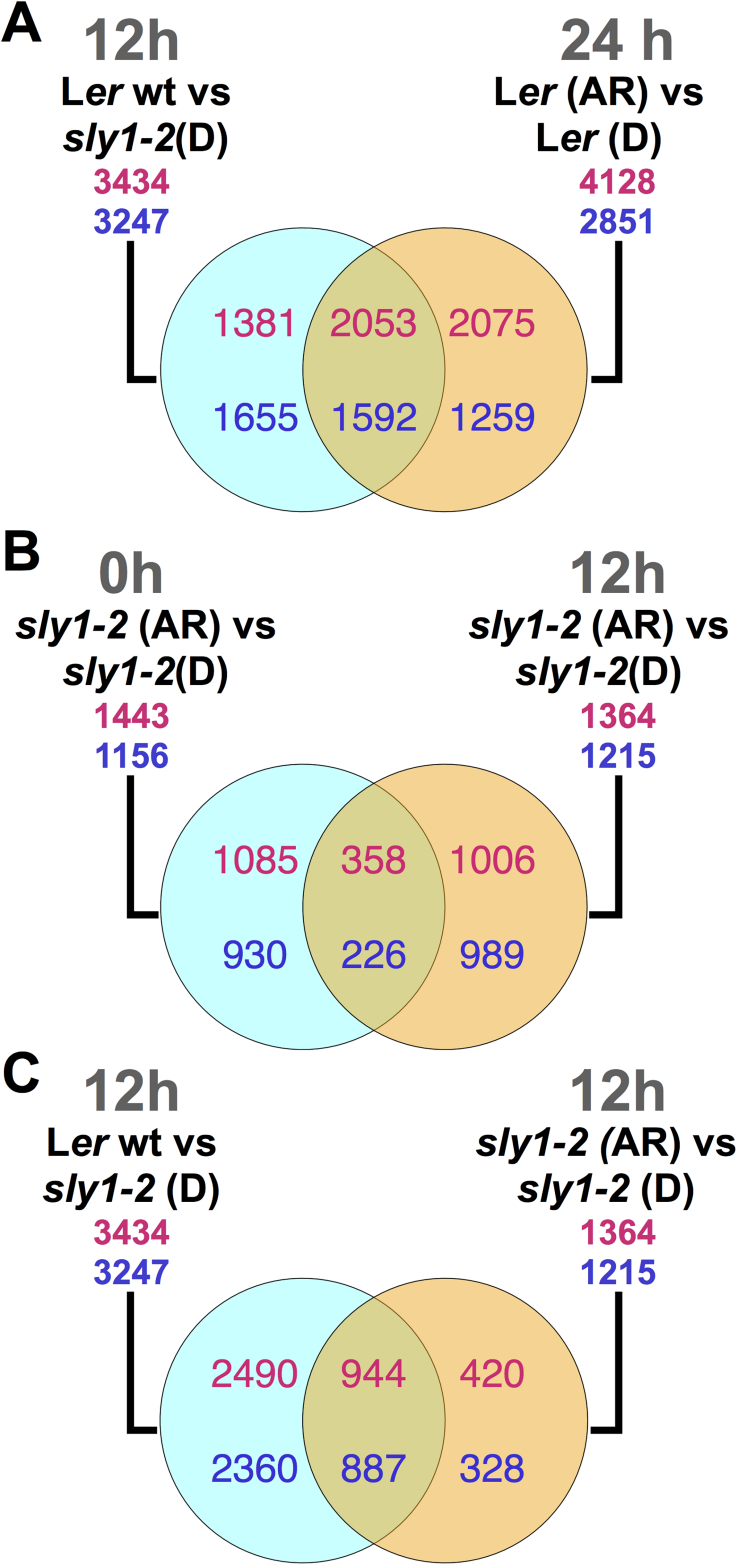
Comparison of differentially-regulated genesets to identify overlaps. (A) Overlap between genes inversely regulated with the *sly1* mutation at 12h with genes differentially-regulated with after-ripening of wild-type L*er* at 24 h (12h WTvsD ∩ 24 h L*er* ARvsD). (B) Overlap between genes AR-regulated genes in *sly1-2* at 0h and at 12h (0h *sly1-2* ARvsD ∩ 12h *sly1-2* ARvsD). (C) Overlap of genes inversely regulated with the *sly1* mutation and with *sly1-2* after-ripening at 12h (12h WTvsD ∩ 12h *sly1-2* ARvsD).

Based on transcriptome profiles, the dormancy and dormancy-loss mechanisms of *sly1-2* mutants have much in common with wild-type dormancy mechanisms. There was more than a 50% overlap between the WTvsD and L*er* ARvsD comparisons in late Phase II (Fig 2A). Thus, many transcripts associated with *sly1-*2 dormancy are also regulated with dormancy loss through after-ripening of Ler wt. This suggests that the transcripts with altered expression may actually cause the dormancy phenotype of *sly1-2*. After-ripening of *sly1-2* was associated with 1385 transcriptome changes similar to those observed in wild-type L*er* (S1B Fig). Moreover, the 86% that overlapped were also differentially abundant in the 12h WTvsD comparison (S1D Fig). These changes occurring with after-ripening of both L*er* and *sly1-2* do not require DELLA destruction, while those that occur only in wild-type L*er* after-ripening likely involve DELLA destruction by SCF^SLY1^ (S2 Table).

**Fig 3:**
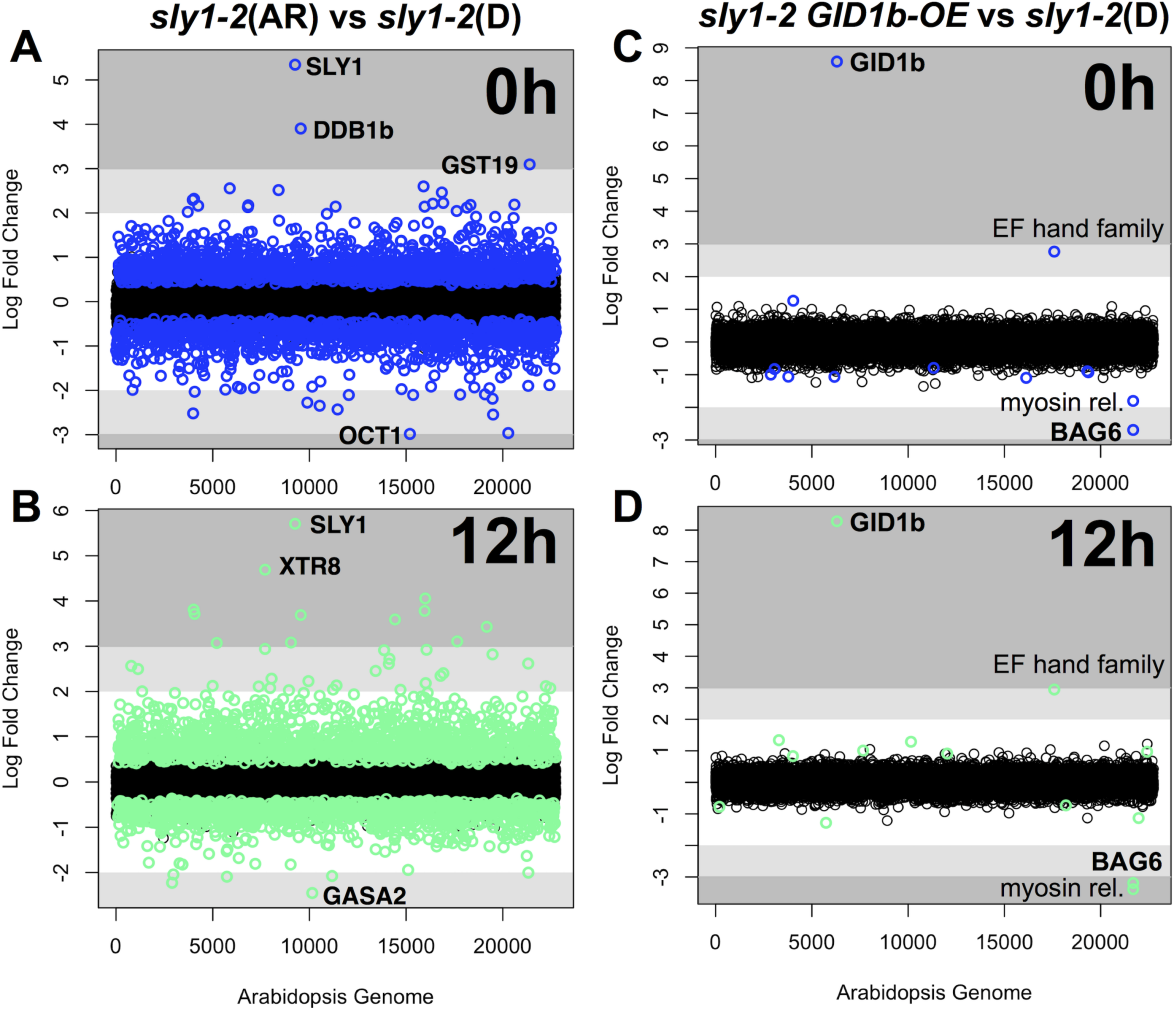
Transcriptome changes with after-ripening or *GID1b-OE*. Genes differentially regulated with *sly1-2* after-ripening at the (A) 0h and (B) 12h imbibition timepoints. Genes differentially regulated with *GID1b-OE* (GIDvsD) at (C) 0h and (D) 12h. Genome-wide expression plots show the skew, magnitude, and chromosomal distribution of differentially-regulated genes. The log_2_-fold changes (logFCs) in transcript abundances for each comparison were plotted on the y-axis versus a number between 1 and 22,810 corresponding to a gene’s chromosomal location on the x-axis. Genes with significant differential regulation are in blue or green (based on FDR p < 0.05). Positive logFC indicates up- and negative logFC down-regulation. Shaded area mark the ±2 and ±3 logFC to allow comparison of magnitude and skew.

AR-dependent transcriptome differences in *sly1-2* (ARvsD) were identified at early (0h) and late (12h) timepoints in Phase II of the germination process (S1 Table). The two imbibition timepoints were transcriptionally distinct. Although a similar number of differentially-regulated transcripts were observed at 0h and 12h in *sly1-2* ARvsD (2599 and 2579, respectively), there was only a 22% overlap between differentially-regulated transcripts at the two imbibition timepoints (Fig 2B). Moreover, analysis of genome-wide expression plots using the adjusted Fisher-Pearson standardized moment coefficient (G_1_) as a measure of skew indicated that the 12h *sly1-2* ARvsD dataset was more highly skewed towards positive changes in expression (G_1_ = 1.19) (Fig 3B), than the 0h *sly1-2* ARvsD dataset (G_1_ = 0.37, where G_1_ = 0 indicates a completely symmetrical dataset) (Fig 3A) [50]. Thus, it appears that more transcripts are induced as germination *per se* approaches.

### Gene ontology analysis of after-ripening and imbibition time

**Fig 4:**
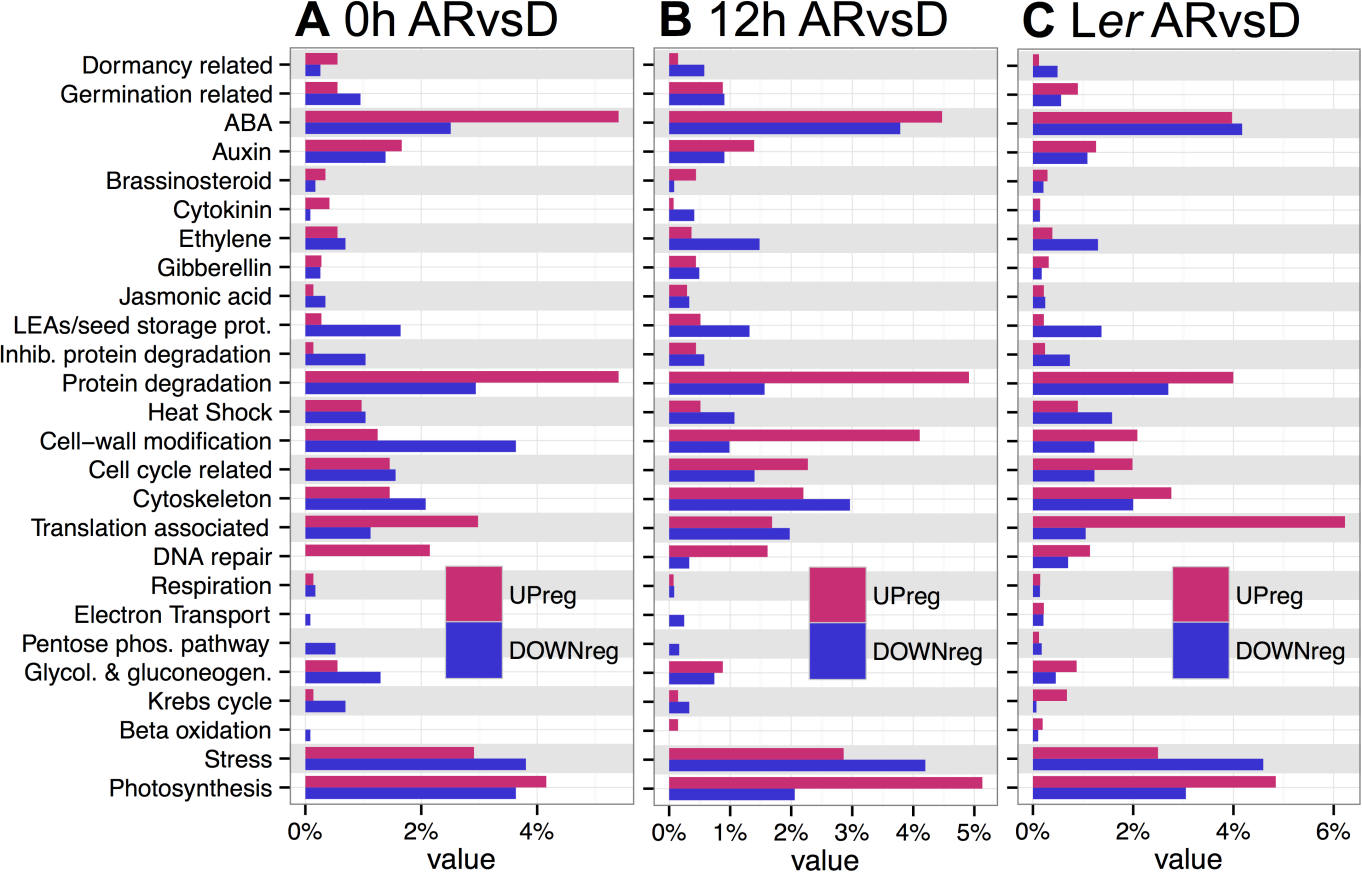
TAGGIT gene ontology analysis of transcriptome changes with after-ripening of *sly1-2* or of wild-type L*er*. (A) Genes differentially regulated with *sly1-2* after-ripening at 0h in early Phase II (*sly1-2* ARvsD comparison), and (B) at 12h in late Phase II. (C) Genes differentially regulated with L*er* after-ripening in late Phase II (24 h L*er* ARvsD). The x-axis value shows the percentage of total up- or down-regulated genes within a dataset.

The gene categories differentially regulated with *sly1-2* after-ripening were examined at the two imbibition timepoints by TAGGIT gene ontology analysis using categories defined by relevance to seed germination [6] (see Materials and Methods; S2 Fig). At 0h, *sly1-2* after-ripening was differentially enriched for categories associated with early Phase II, including DNA repair, protein degradation/inhibition of protein degradation (involved in mobilization of seed storage proteins), and protein translation (Fig 4A). At 12h, *sly1-2* after-ripening was differentially enriched for categories associated with late Phase II and the transition to Phase III, including stored reserve mobilization (beta-oxidation, glycolysis and gluconeogenesis, protein degradation), cell division, cell-wall modification (needed for seedling emergence and growth), and photosynthesis (Fig 4B). The TAGGIT profile at 12h resembled late Phase II of the L*er* ARvsD (Fig 4C). Transcriptome changes in 24 h L*er* ARvsD accounted for 54% of those in *sly1-2* ARvsD at the 12h, but only 28% of those at the 0h timepoint (S1B Fig).

**Fig 5:**
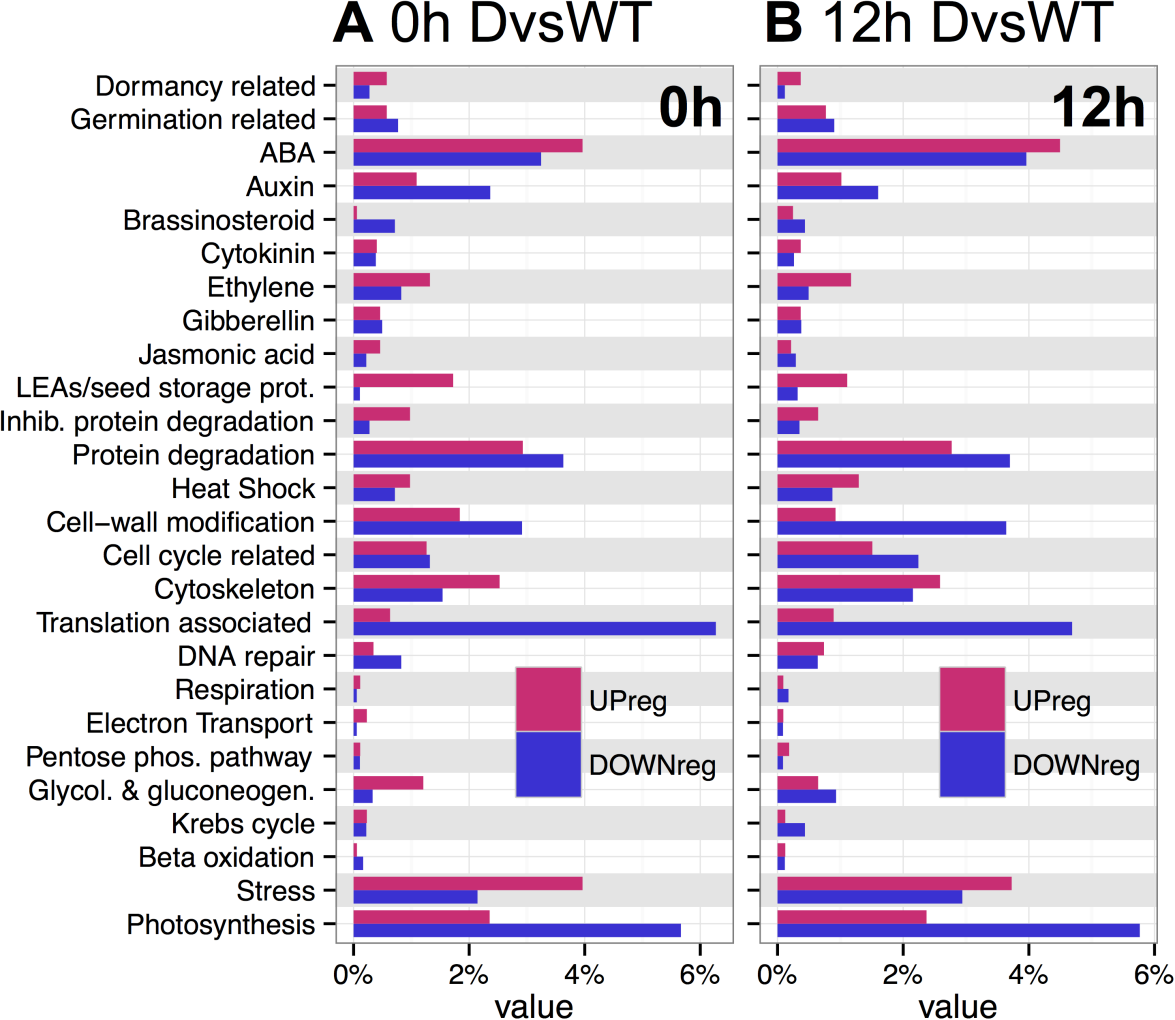
TAGGIT gene ontology analysis of DvsWT transcriptome changes in seeds. (A) At 0h in early Phase II. (B) At 12h in late Phase II. The value on the x-axis shows the percentage of either the total up-regulated or total down-regulated genes within a dataset.

The TAGGIT category with the most distinct difference between *sly1-2* and wild-type was the protein translation category. Protein translation was more highly up-regulated in the 24h L*er* ARvsD than at either of the *sly1-2* ARvsD timepoints (Fig 4C). Consistent with the notion that *SLY1* is needed for translation-associated gene induction, TAGGIT analysis of the DvsWT dataset revealed down-regulation of the translation-associated category in *sly1-2* at 0h and 12h (Fig 5A and 5B). Translation-associated genes down-regulated in the DvsWT and up-regulated in the L*er* ARvsD comparisons included ribosomal structural proteins, tRNA synthetase genes, and elongation factors, as shown in S3 Table. Thus, *SLY1* appears to be needed for the efficient up-regulation of genes involved in protein translation with after-ripening. If so, then we would expect GA to regulate translation-related gene expression. Indeed, our TAGGIT analysis revealed that protein translation is the most strongly GA-upregulated and DELLA-downregulated gene category in early Phase II seeds (S3A, S3B and S3C Fig; [49]).

**Fig 6:**
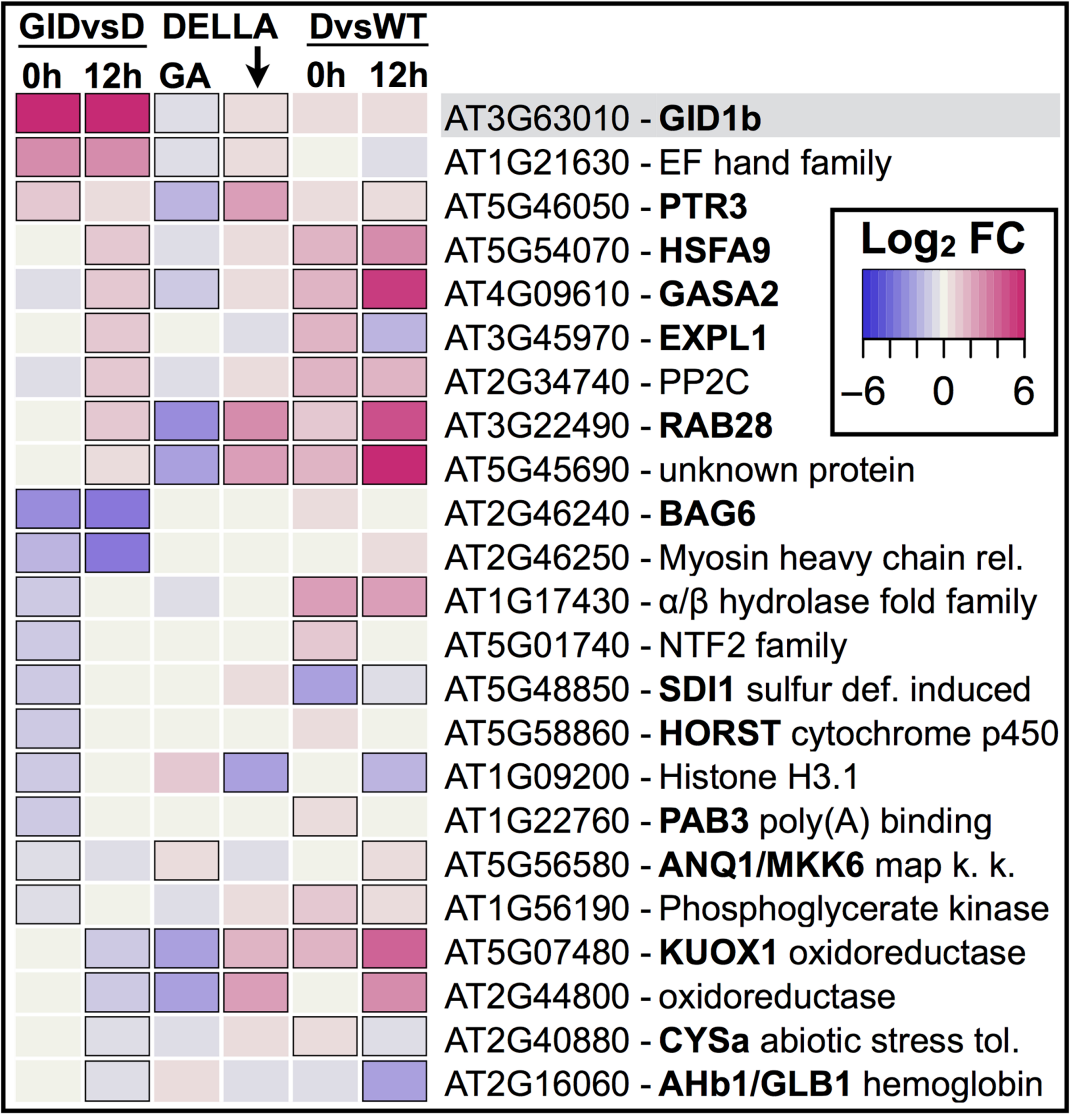
Differentially-regulated genes with rescue of *sly1-2* germination by *GID1b*-overexpression. The GIDvsD column provides the *GID1b-OE*–UP (red) and -DOWN (purple) regulated genes at 0h and 12h. The GA column indicates GA-regulation based on the L*er* wt vs *ga1-3* comparison and DELLA column indicates DELLA-regulation based on the *ga1-3* vs *ga1-3 4x della* comparison (Cao et al. [49]). The DvsWT column indicates the DELLA/*sly1*-regulation. Black borders indicate significance based on an FDR of p < 0.05.

Some categories switched from being more up- to more down-regulated (or vice versa) between the 0h and 12h timepoints, suggesting that negative regulation of germination *per se* early in Phase II may be reversed in late Phase II. For example, the dormancy-related category was more up-regulated, while the germination and protein degradation categories were more down-regulated at 0h than at 12h. The ABA category was differentially enriched at both timepoints, but showed a greater ratio of up- to down-regulation at 0h. Note, however, that the TAGGIT ABA category does not differentiate between positive and negative regulators. There was strong down-regulation in the ethylene category at the 12h timepoint, but little change in the GA-related category. The L*er* ARvsD comparison also showed little change in the GA category, suggesting that this was not due to the *sly1-2* mutation (Fig 4C; [6]).

### After-ripening regulated transcription factor targets

Transcription factors (TFs) possibly controlling after-ripening-regulated transcription were identified in the *sly1-2* ARvsD datasets using the Plant GeneSet Enrichment Analysis (PlantGSEA) tool that identifies TFs based on enrichment of their experimentally identified targets in a differentially-regulated gene set (Table 1; [51–53]). There was strong enrichment for E2Fa–DPa (the heterodimeric E2Fa-*D**roso**p**hila melanogaster* a complex) and PIF1/PIL5 targets at both 0h and 12h. E2Fa–DPa may promote germination *per se* because it is a positive regulator of the cell division cycle, and stimulates gene expression involved in DNA replication, nitrate assimilation, and cell wall biosynthesis [54,55]. PIF1/PIL5 is a light-repressed negative regulator of seed germination, and was enriched in the down-regulated fraction [56,57]. There was also enrichment for targets of AP2 (APETALA2), and light-responsive TFs, PIF3 and HY5 (ELONGATED HYPOCOTYL5) [58]. In addition to floral development, AP2 regulates seed size, seed coat formation, and storage of seed mucilage during development [59]. Interestingly, PIF1/PIL5 and PIF3 are DELLA-interacting proteins, raising the possibility that DELLA-regulated genes are after-ripening-regulated via PIF1 and PIF3.

**Table 1:** Analysis of *sly1-2* after-ripening-regulated genes for enrichment of transcription factor targets.

TF	Description	0h		adj p-val^a^	12h		adj p-val^a^
**E2Fa-DPa**	cyclin D/retinoblastoma pathway (cell cycle)	**DOWN**	All	6.61×10^−04^	**UP**	All	8.60×10^−12^
			Conf.	—		Conf.	—
**HY5**	bZIP TF, light regulated, mediates ABA responses	**DOWN**	All	1.07×10^−03^			
			Conf.	4.03×10^−03^			
**AP2**	Ethylene responsive TF (floral identity)	**DOWN**	All	0.0152	**UP**	All	1.54×10^−03^
			Conf.	0.0152		Conf.	1.54×10^−03^
**PIF1/PIL5**	bHLH, negatively regulates germination in dark	**DOWN**	All	2.64×10^−03^	**DOWN**	All	7.88×10^−16^
			Conf.	0.0212		Conf.	8.57×10^−16^
**PIF3**	negative regulator of phyB signaling	**DOWN**	All	0.0562			
			Conf.	0.0562			

Conf., Confirmed, includes targets with function shown by two or more approaches with *in vivo* evidence; All, targets shown by one or more approach, including confirmed targets.

^a^Significance was determined based on a Fisher statistical test with Yekutieli (FDR under dependency) adjustment (p < 0.06).

Zentella et al. [24] identified 18 putative DELLA RGA targets by microarray, and confirmed DELLA localization to 8 of these promoters by DELLA ChIP-qPCR. We examined whether these 18 putative DELLA target genes were differentially regulated by after-ripening, *sly1*, GA, or DELLA (Table 2). Our reanalysis of the Cao et al. [49] microarray datasets defined GA-regulated transcripts based on the WT vs *ga1-3* comparison, and DELLA-regulated transcripts based on *ga1-3* vs *ga1-3 della* (Fig 1C). For convenience, transcripts down-regulated in the presence of GA (lower in WT than *ga1-3*) are called GA-DOWN, and those up-regulated by DELLA (higher in *ga1-3* than in the *ga1-3 della*) are called DELLA-UP. DELLA-UP transcripts are generally also GA-DOWN [24,49]. Since *sly1-2* mutants over-accumulate DELLA, DELLA-UP genes are expected to be up-regulated in *sly1-2* (*sly1*-UP), and vice versa. Indeed, out of the 3213 DELLA-regulated and the 3559 0h *sly1*-regulated genes, only 72 genes showed opposite DELLA and *sly1*-regulation. If *sly1-2* after-ripening leads to down-regulation of DELLA by a non-proteolytic mechanism, then we would expect DELLA-UP genes to be after-ripening (AR)-DOWN, and vice versa. Of the 18 putative DELLA targets, 8 showed significant differential AR-regulation, and of these 6 AR-DOWN targets were also DELLA-UP and/or *sly1*-UP at 12h. Moreover, the DELLA target *GID1a* was AR-DOWN/DELLA-UP at 0h and 12h. The DELLA-UP target *XERICO* was AR-UP at 0h and AR-DOWN at 12h of imbibition, suggesting that its regulation changes over imbibition time. The MYB TF *At3g11280* was DELLA-UP and AR-UP.

**Table 2:** *sly1-2* after-ripening-, GA-, and DELLA-regulation of putative DELLA targets.

		ARvsD^b^			WT vs *ga1*^c^	*ga1* vs *ga1 della* ^c^			DvsWT^d^		
AGI locus^a^	Name	0h	12h	AR^e^			GA^e^	DELLA^e^	0h	12h	*sly1*^e^
**At3g11280**	MYB TF	2.14	0.67	UP	-0.60	0.54	DOWN	UP	—	—	
**At3g05120**	GID1b	—	—		-0.37	0.38	DOWN	UP	—	—	
**At2g04240**	XERICO	0.65	-0.55	UP/DOWN	-3.18	2.63	DOWN	UP	1.65	3.13	UP
**At3g05120**	GID1a	-0.71	-1.28	DOWN	-1.92	1.71	DOWN	UP	1.50	3.03	UP
At5g51810	GA20ox2	—	-1.20	DOWN	-5.00	3.79	DOWN	UP	1.64	5.27	UP
At4g23060	IQD22	—	-0.92	DOWN	—	—			—	0.80	UP
**At1g50420**	SCL3	—	-0.81	DOWN	-3.45	2.84	DOWN	UP	1.84	4.27	UP
At3g52870	CaM-BP	—	-0.54	DOWN	—	—			—	1.31	UP
**At1g67100**	LBD40	—	-0.52	DOWN	-2.20	1.97	DOWN	UP	0.50	2.77	UP
At1g15550	GA3ox1	—	—		-1.31	1.57	DOWN	UP	—	0.86	UP
**At5g50915**	bHLH137	—	—		—	—			—	—	
At2g31730	bHLH154	—	—		—	—			—	-0.68	DOWN
**At5g52830**	WRKY27	—	—		—	—			—	—	
At5g67480	BT4	—	—		-1.81	1.48	DOWN	UP	1.06	2.57	UP
At2g45900	Exp-PT1	—	—		-0.93	0.95	DOWN	UP	0.89	1.22	UP
At2g34340	Exp-PT2	—	—		—	—			—	0.82	UP
At4g36410	UBC17	—	—		—	—			—	—	
At4g19700	RING	—	—		—	—			—	—	

^a^Putative DELLA targets identified in Zentella et al. [24]; targets significantly enriched by ChIP-qPCR indicated in **bold**.

^b^*sly1-2*(AR) vs *sly1-2*(D).

^c^Dataset from Cao et al. [49], Comparisons: L*er* wt vs *ga1-3* and *ga1-3* vs *ga1-3 della*.

^d^*sly1-2*(D) vs WT (L*er*).

^e^AR: after-ripening-regulated, GA: GA-regulated, DELLA: DELLA-regulated, *sly1*: *sly1*-regulated.

### Few transcripts are differentially regulated with *GID1b* overexpression, and a subset of these are DELLA-regulated

Although *GID1b-OE* rescued *sly1-2* germination as well as after-ripening, it was associated with far fewer changes in transcript accumulation, including only 23 transcripts over the 0h and 12h timepoints (Fig 6; S4 Fig; S1 Table). In the GIDvsD comparison, there were 13 differentially-regulated genes at 0h and 14 at 12h. Four genes showed the same differential regulation at both imbibition timepoints, including *GID1b*, *At1g21630*, *BAG6* (*BCL-2-Associated Anthogene6*), and *At2g46250*. Given that it is overexpressed on the 35S promoter, *GID1b* served as an internal control, showing the strongest up-regulation in GIDvsD at 0h and 12h (logFC > 8, Fig 3C and 3D). Down-regulated genes *BAG6* (*At2g46240*) and *At2g46250*, may share a promoter as they are oriented head-to-tail on chromosome 2. The putative EF hand domain protein *At1g21630* was *GID1b*-UP at 0h and 12h in both the GIDvsD and *sly1-2* GIDvsAR comparisons (logFC > 2.68).

In the non-proteolytic GA signaling model, *GID1b* overexpression rescues germination by down-regulating DELLA repressors via protein-protein interaction [41,42]. Thus, *GID1b*-*OE* was expected to mainly cause changes in DELLA-regulated transcript levels. Indeed, only 3 of the 22 *GID1b-OE*-regulated genes were not found to be GA-, DELLA-, or *sly1*-regulated (Fig 6; S4 Fig). It was expected that *GID1b*-*OE*-UP genes would also be GA-UP/DELLA-DOWN/*sly1*-DOWN or vice versa. Surprisingly, 15 of the 22 *GID1b-OE*-regulated gene showed the opposite expression pattern, suggesting that the change with *GID1b-OE* may not be due to lifting DELLA repression (Fig 6). The *EXPL1* gene behaved as expected, showing *GID1b-OE*-UP and *sly1*-DOWN regulation at 12h. Six of the *GID1b-OE*-DOWN genes behaved as expected, showing GA-DOWN/DELLA-UP or *sly1*-UP regulation. Of these, the putative oxidoreductases *At5g07480* (*KUOX1*) and *At2g44800* are MYB-type TF GL1 targets, while putative nuclear transport factor 2 family member *At5g01740* is a TF GL3 target based on PlantGSEA. Both GL1 and GL3 are known DELLA-interacting proteins [29], supporting the notion that *GID1b-OE* may alter expression of these genes by down-regulating DELLA.

### Comparison of after-ripening and *GID1b-OE* differentially regulated transcripts

**Fig 7:**
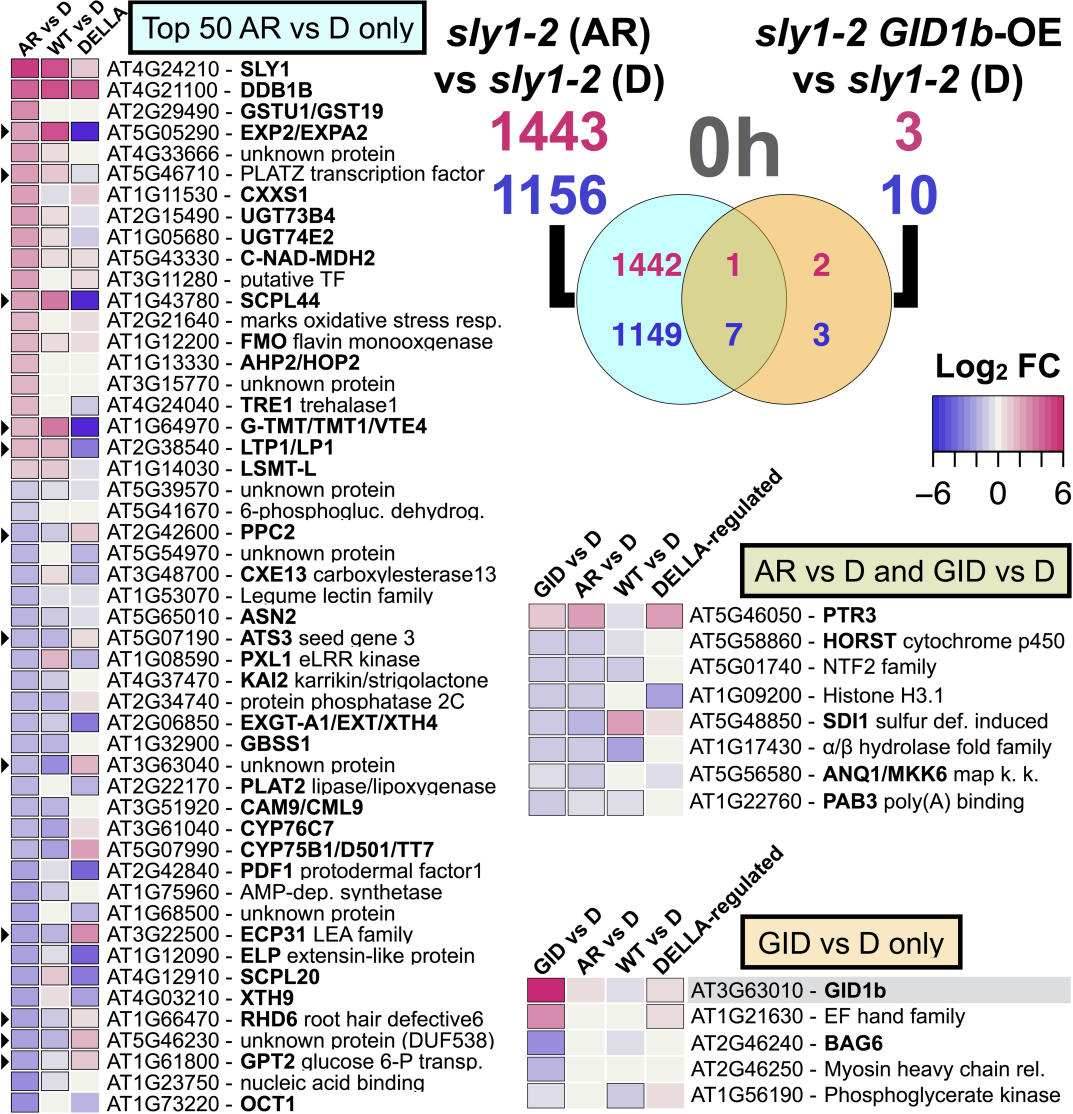
Comparison between after-ripening- and *GID1b-OE-* regulated transcriptome changes at 0h. Overlap between *sly1-2* AR-regulated genes with *GID1b-OE*-regulated genes at 0h (0h *sly1-2* ARvsD ∩ 0h GIDvsD) is shown in a Venn diagram. The three heatmaps correspond to the three areas of the Venn diagram: the top 50 AR-regulated genes that were not *GID1-OE*-regulated (light blue), the 5 *GID1-OE*-regulated genes that were not AR-regulated (orange), and the 8 *GID1b-OE*-regulated genes that were AR-regulated (brown). Up-regulation is red, down-regulation purple, and significant differences are marked with a black border (FDR of p < 0.05). The ARvsD columns indicate *sly1-2* ARvsD and the DELLA/DELLA-regulated columns indicate DELLA-regulation based on the *ga1-3* vs *ga1-3 4x della* comparison from Cao et al. [49] (0h timepoint). Black triangles indicate significant regulation in the expected direction if *sly1-2* after-ripening occurs through negative regulation of DELLA.

**Fig 8:**
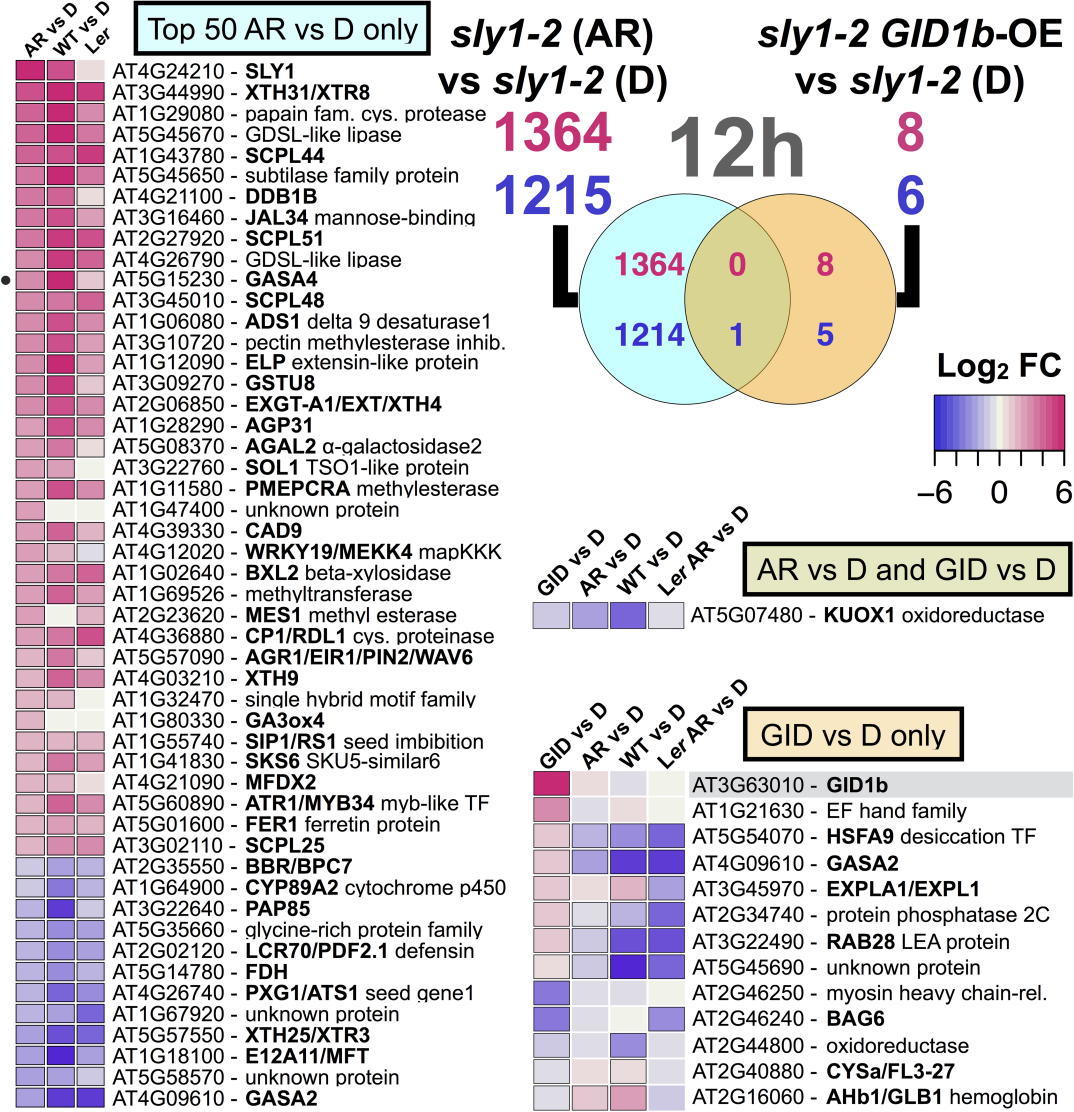
Comparison between after-ripening- and *GID1b-OE-* regulated transcriptome changes at 0h. Overlap of *sly1-2* AR-regulated with *GID1b-OE*-regulated genes at 12h (12h *sly1-2* ARvsD ∩ 12h GIDvsD) is shown in a Venn diagram. The L*er* ARvsD and L*er* columns indicate the 24 h L*er* ARvsD comparison from Carrera et al. [6]. The black circle marks *GASA4*, a 12h *GID1b-OE*-regulated transcript based on RT-qPCR.

Since long after-ripening and *GID1b-OE* both rescue *sly1-2* germination without DELLA destruction, we examined whether they were associated with similar transcriptome changes. Many more genes were differentially regulated with after-ripening than with *GID1b-OE* (Fig 3A, 3B, 3C and 3D). More of the *GID1b-OE*-regulated transcripts (GIDvsD) showed similar AR-regulation (*sly1-2* ARvsD) at the 0h than at the 12h imbibition timepoint. Of the 13 *GID1b-OE*-regulated genes at 0h, 1 *GID1b-OE*-UP and 7 *GID1b-OE*-DOWN genes showed similar AR-regulation (Fig 7). Of the 14 *GID1b-OE*-regulated genes at 12h, only 1 *GID1b-OE*-DOWN gene (*KUOX1*) showed similar AR-regulation (Fig 8). This gene was also down-regulated in 12h WTvsD and in the 24 h L*er* ARvsD comparison, suggesting that it is AR-regulated in both wild-type and *sly1-2* [6]. Thus, it appears that there is more similarity in early Phase II than as the seeds approach germination *per se*.

We expected to find agreement between comparisons of lines that germinate to lines that do not germinate in late Phase II, including the 12h GIDvsD, the 12h WTvsD, and both the 12h *sly1-2* ARvsD and 24 h L*er* ARvsD comparisons (Fig 8). Indeed, all of the 50 most differentially-regulated genes in the 12h *sly1-2* ARvsD comparison showed similar differential regulation in the 12h WTvsD, and all but 1 with 24 h L*er* ARvsD. In contrast, most of the 12h *GID1b-OE*-regulated genes did not show similar regulation in WTvsD, or with after-ripening of *sly1-2* and L*er*. In fact, 7 showed regulation in the opposite direction. For example, *HSFA9*, *GASA2*, *At2g34740*, *RAB28*, and *At5g45690*, were *GID1b-OE*-UP but down-regulated in 12h *sly1-2* ARvsD, 12h WTvsD, and 24 h L*er* ARvsD. Additionally, *GASA4* and *AHb1* (*A**rabidopsis nonsymbiotic* *H**emoglo**b**in1*) were *GID1b-OE*-DOWN, but up-regulated in 12h WTvsD, and with *sly1-2* after-ripening. Thus, many of the 12h *GID1b-OE* differentially-regulated genes actually showed opposite regulation from other germinating versus non-germinating comparisons. This may suggest a negative feedback response.

### Comparison of RT-qPCR and microarray analyses of gene expression

**Fig 9:**
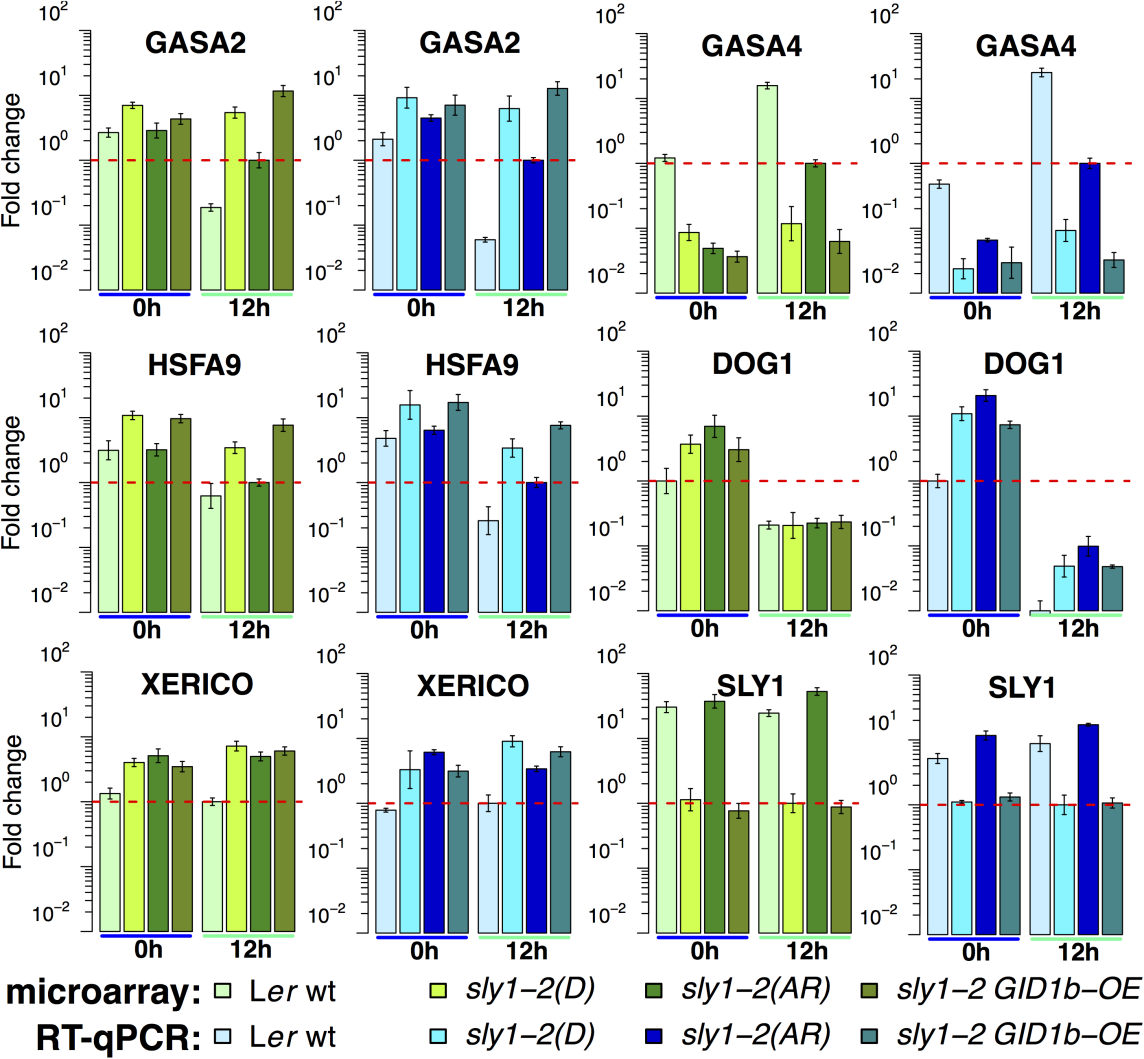
Comparison of transcriptome changes measured by microarray and RT-qPCR analyses. For each gene, relative expression in microarray (blue) and RT-qPCR (green) analyses are shown relative to the same calibrator, set to a height of 1 (10^0^) and indicated by the red dotted-line. RMA-normalized microarray data was analyzed using the ddCT method relative to the same constitutively expressed HBT control gene used for the ddCT analysis of RT-qPCR data. Statistical significance was determined by pairwise t-test with Bonferroni-Holm correction for multiple comparisons (see S6 Fig for p-values). Error bars represent SD.

RT-qPCR was conducted to validate microarray results using six genes differentially regulated in multiple comparisons (Fig 9). For comparison, both RT-qPCR and microarray expression were plotted relative to *HBT* (*HOBBIT*, *At2g20000*), a constitutive control gene [60]. There appeared to be a high degree of similarity and a linear correlation between the RT-qPCR results and the microarray results, although some differences were better observed by RT-qPCR than by microarray (S5 Fig; Fig 9). As in L*er* ARvsD, *GASA2* and *HSFA9* were AR-DOWN both in the 12h *sly1-2* ARvsD microarray and RT-qPCR analyses. RT-qPCR also confirmed differential regulation of *GASA2* and *HSFA9* by *GID1b-OE* at 12h (Fig 9; S6 Fig).

Based on a previous RT-PCR analysis in *sly1-2* seeds imbibed for 24 h following cold stratification, *XERICO* and *GASA4* were expected to be AR-regulated at 12h [21,42]. Indeed, *XERICO* was significantly AR-DOWN and *GASA4* AR-UP in *sly1-2* ARvsD at 12h based on microarray and RT-qPCR (Fig 9; S6 Fig). Moreover, *GASA4* was significantly AR-UP (logFC = 1.05; p = 1.26 x 10^−4^) and *XERICO* significantly AR-DOWN (logFC = -1.32; p = 6.14 x 10^−3^) in the L*er* ARvsD comparison [6]. A previous analysis detected *GID1b-OE*-down-regulation of *XERICO* and *GASA4* at 24 h of imbibition following cold stratification [42]. Although these genes were not significantly *GID1b-OE-*regulated in our microarray study based on an FDR adjusted-p-value < 0.05, they both exhibited a downward trend at the 12h timepoint. By RT-qPCR analysis at 12h, *XERICO* showed a non-significant downward trend, whereas *GASA4* was significantly *GID1b-OE*-DOWN (p = 2.9 x 10^−3^; Fig 9; S6 Fig). Thus, *GASA4* was added to the short list of *GID1b-OE-*regulated transcripts.

In previous studies, *DOG1* (*DELAY OF GERMINATION1*) expression levels were positively correlated with higher seed dormancy [61,62]. Consistent with this, *DOG1* mRNA levels were lower with L*er* after-ripening (logFC = -0.91, p = 0.02), and higher in dormant *sly1-2* than in WT at 0h (Fig 9; S6 Fig). *DOG1* was not down-regulated with *sly1-2* after-ripening or with *GID1b-OE*. RT-qPCR analysis showed that *DOG1* mRNA levels were AR-UP in *sly1-2* at 0h (logFC = 0.93, p = 0.05). Although *DOG1* expression at 12h was very low, a similar expression pattern was observed by RT-qPCR.

The gene with the largest fold increase with after-ripening was *SLY1* (logFC: 5.70 (0h), 5.34 (12h); Figs 7 and 8). This was consistent with RT-PCR analysis and previous work (Fig 9; [63]). Note that the 2-bp deletion in the *sly1-2* transcript did not interfere with detection by RT-PCR primers or by the 11 *SLY1* Affymetrix ATH1 probes. The only gene with a *SLY1*-like expression pattern was *DDB1b*, a subunit of the ultraviolet-damaged DNA-binding protein complex (logFC: 3.90 (0h), 3.69 (12h)) [64]. Both *SLY1* and *DDB1b* were more highly expressed in WT and in *sly1-2*(AR) than in *sly1-2*(D), suggesting that *SLY1* and after-ripening stimulate expression of these genes. Both of these transcripts were DELLA-UP (Fig 7; [49]).

## Discussion

The mechanisms governing seed dormancy release remain one of nature’s most interesting mysteries. Genome-wide transcriptional assays in *sly1-2* were designed to ask specific questions about DELLA-regulated and DELLA-independent seed dormancy loss. Although *sly1-2* cannot degrade DELLA repressors via the ubiquitin-proteasome pathway, non-proteolytic GA signaling allows some rescue of seed dormancy by long after-ripening or by *GID1b* overexpression [42]. Comparing *sly1-2* ARvsD to L*er* ARvsD revealed that over half of the transcriptome changes with after-ripening depend upon DELLA destruction (S1B and S1D Fig; Fig 4; [6]). Furthermore, TAGGIT analysis revealed a major loss in abundance of transcripts associated with protein translation in the *sly1-2* mutant (Fig 5). The GIDvsD comparison revealed that the rescue of *sly1-2* germination can occur with remarkably few changes in gene transcript levels (Figs 3 and 6). Within this small number of changes, after-ripening and *GID1b-OE* appear to rescue *sly1-2* germination by only partly overlapping transcriptional mechanisms (Figs 7 and 8). *GID1b-OE* differentially-regulated genes overlapped with AR-regulated genes more in early than in late Phase II. Many DELLA-regulated genes were inversely regulated with after-ripening and *GID1b*-overexpression.

### After-ripening resulted in both *SLY1* and DELLA-proteolysis dependent and independent transcriptome changes

We know that *SLY1* and DELLA destruction play a role in after-ripening because the *sly1-2* single gene mutation causes a large change in the imbibing seed transcriptome associated with a drastic shift in the time required for dry after-ripening from 2 weeks to around 2 years. Genes associated with seed dormancy due to DELLA overaccumulation were identified in the DvsWT transcriptome comparison. Over half of these *sly1*-regulated genes were oppositely regulated with after-ripening of L*er* or *sly1-2*, supporting the role of DELLA in seed dormancy and its release (Fig 2A and 2C; [6]).

Although *sly1-2* mutants cannot degrade DELLA protein, GA treatment can cause up-regulation of GA-stimulated gene expression [42]. A similar fraction of late Phase II AR-regulated genes in *sly1-2* (35%) or wild-type L*er* (33%) were known GA-regulated genes, in spite of the larger total number of transcriptome changes in L*er* (S7A and S7B Fig; [49]). Thus, some GA-regulated transcripts do not require *SLY1* and associated DELLA destruction to respond to after-ripening.

If after-ripening and *GID1b-OE* are rescuing *sly1-2* seed germination at least in part through DELLA-regulated transcriptional changes, then we might expect to identify known targets of DELLA-interacting or GA-regulated transcription factors in our differentially regulated gene sets. Indeed, enrichment analysis for transcription factor targets within after-ripening-regulated genes in *sly1-2* identified: 1) the DELLA-interacting TFs, PIF1/PIL5 and PIF3, 2) the GA- and light-regulated TF, HY5 (Table 1; [58,65,66]). HY5 is active in early Phase II and is a positive regulator of ABA responses during seed germination through *ABI5* [67–69]. Among AR-regulated genes, there were also 54 and 127 known targets of the DELLA interacting transcription factors GL1 and GL3, respectively (S4 Table). GL1 and GL3 interact with DELLA to regulate jasmonate (JA) and GA signaling [29]. Given evidence suggesting that JA signaling stimulates dormancy loss and germination in barley and wheat, future work should examine whether GL1 and GL3 mediate crosstalk between GA and JA signaling during seed germination [70,71].

### Gene categories associated with after-ripening

Although both dormant and after-ripened seeds enter Phase II, TAGGIT analysis suggests that what prevents dormant seeds from germinating may be AR-regulation of genes associated with early and late Phase II processes at 0h and 12h, respectively (Fig 4A and 4B). Thus, seed dormancy may block germination in part by preventing the accumulation of transcripts involved in Phase II processes. Changes in similar transcript categories were associated with after-ripening of *sly1-2* and L*er* wt, suggesting that *sly1-2* undergoes a “natural” after-ripening process (S1B Fig; [6]).

TAGGIT gene ontology analysis suggested that after-ripening upregulates protein translation, and that this upregulation involves GA signaling. After-ripening resulted in massive up-regulation of protein translation-associated genes in late Phase II in wild-type L*er*, but not in *sly1-2* (Fig 4). Additionally, comparison of 2 week after-ripened *sly1-2* and wild-type showed similarly massive down-regulation of the same category in *sly1-2* in both early and late Phase II (Fig 5). Furthermore, our re-analysis of GA- and DELLA-regulated genesets from Cao et al [49] showed that translation-associated genes were the most highly regulated category. This suggests that GA signaling and *SLY1* are required for the efficient induction of protein translation-associated genes in germinating seeds. Future work should examine whether the failure of GA biosynthesis and signaling mutant germination is associated with a failure in protein translation. This notion is interesting given that inhibition of protein translation with cyclohexamide fully blocked germination, whereas inhibition of *de novo* transcription by α-amanitin slowed seed germination [72]. Thus, proteins translated from stored mRNAs or mRNAs transcribed early in seed imbibition (before α-amanitin uptake) appear to be sufficient for germination *per se*. Investigation of the stored mRNA transcriptomes in dry seeds or of the translatome in early imbibition may shed light on key proteins involved in seed germination.

### Reduced *sly1* dormancy due to *GID1b-OE* was associated with few transcriptome changes

Very few transcriptional changes occurred with *GID1b-OE* in *sly1-2* while many changes occurred with *sly1-2* after-ripening (Fig 3A, 3B, 3C and 3D). This result indicates that a major change in *sly1-2* seed germination potential can occur with few changes in transcript levels. There are three possible explanations: 1) *GID1b-OE* rescues *sly1-2* germination via a non-transcriptional mechanism, 2) *GID1b-OE* rescue of *sly1-2* germination results from transcriptional changes during seed development rather than during seed imbibition, or 3) the 22 *GID1b-OE*-regulated transcripts are particularly critical regulators of dormancy loss. While future work will need to examine whether loss or gain of *GID1b-OE-*regulated gene function alters seed germination potential, it is interesting to consider the functional categories of these *GID1b-OE*-regulated transcripts.

Several of the *GID1b-OE*-regulated transcripts encode ABA-responsive seed-related regulatory genes (Fig 6). The TF *HSFA9*, and the LEA (Late Embryogenesis Abundant protein) *RAB28* were *GID1b-OE*-up-regulated. Both play roles in seed desiccation tolerance and are regulated by the seed-specific TF ABI3 of the ABA signaling pathway [73–77]. Consistent with its up-regulation, overexpression of *RAB28* increased germination rates under both standard and osmotic stress conditions [78]. *HSFA9* is not heat stress-responsive, and is the only one of 21 Arabidopsis HSFs that is solely expressed in seeds starting late in seed development [74]. HSFA9 also regulates seed longevity. Another *GID1b-OE*-up-regulated transcript, *At2g34740*, encodes a protein phosphatase type 2C (PP2C). Although not well characterized, *At2g34740* was induced by ABA and repressed in the ABA-signaling SNF1-related protein kinase 2 (SnRK2) gene triple mutant, *srk2D srk2e srk2i* [79]. This suggested a possible role in ABA signaling during seed development or germination [80,81].

Both nitric oxide (NO) and reactive oxygen species (ROS) have been implicated in stimulating seed germination (reviewed in [82]). NO regulates seed oxygen levels during germination, but also acts as a signaling molecule to promote seed germination [83]. NO appears to promote DELLA protein accumulation and represses *SLY1* expression in seedlings [84]. The *GID1b-OE*-down-regulated non-symbiotic hemoglobin *AHb1* modulates NO accumulation in seeds and regulates expression of ROS metabolism and ABA signaling genes [85,86]. ROS likely stimulates seed germination through endosperm weakening and promotion of programmed cell death in the aleurone, but ROS accumulation also leads to oxidative damage. In total, 6 of the 15 *GID1b-OE*-down-regulated genes were implicated in oxidative stress regulation, including *GASA4*, *AHb1*, *BAG6*, *CYSa*, *KUOX1*, and the oxidoreductase *At2g44800* [86–89]. Oxidation of specific RNAs and protein disulfide bonds may play a functional role in dry after-ripening [9,90–92]. Moreover, germinating seeds need protection from oxidative damage as metabolism is activated (reviewed in [2], [93]).

We also found GA-regulated or signaling genes among the *GID1b-OE*-regulated transcripts (Fig 6). PTR3 is a GA transporter with homology to peptide transporters, and is likely involved in stress tolerance during germination [94–96]. *PTR3* was *GID1b-OE*- and AR-up-regulated in early Phase II. GA promotes cell elongation by inducing cell wall loosening enzymes such as the *GID1b-OE*-UP expansin family member, *EXPL1* [97–99]. Two members of the GA-regulated GASA gene family, *GASA2* and *GASA4*, were oppositely regulated with *GID1b-OE* and after-ripening (Fig 9). The fact that *GASA4* is *sly1*-down and AR-upregulated is consistent with the fact that *GASA4*-overexpression promotes seed germination. Since multiple GASAs block germination, it is possible that *GASA2* is *sly1*-UP and AR-DOWN because it negatively regulates germination [89,100].

One of the fastest GA responses is an increase in cytosolic calcium [101]. The calcium signaling gene *BAG6* (a putative calmodulin-binding protein) was *GID1b-OE*-DOWN, while the calcium-binding EF hand family protein (*At1g21630*) was one of the most strongly *GID1b-OE*-UP transcripts (Fig 3). Future work should examine whether GA acts via calcium signaling in the cytosol to stimulate seed germination. This notion is credible because: 1) the rice and Arabidopsis GID1 proteins localize both to the nucleus and to the cytoplasm, and 2) Arabidopsis *GID1a* was shown to stimulate GA signaling both when altered to localize solely to the nucleus and solely to the cytoplasm [39,102,103].

The idea of a non-transcriptional mechanism of GA signaling is interesting given that gene ontology showed relatively few changes in GA-related transcripts associated with after-ripening of *sly1-2*, wild-type L*er*, and of barley (Fig 4A, 4B and 4C; [6,70]). In the barley aleurone, GA signaling via a calcium-dependent protein kinase in the cytosol occurs independently of nuclear GA signaling [104,105]. The idea that some GID1-mediated GA signaling may not involve direct transcriptional regulation suggests a DELLA-independent mechanism of GA signaling, since DELLA is a nuclear-localized protein believed to function entirely through transcriptional regulation [19,36,106,107]. Future studies should examine if *GID1b-OE* rescue of *sly1* germination requires the ability to physically interact with DELLA protein.

### Similarity of *GID1b-OE*- and AR-regulation in *sly1-2* depends on imbibition time

The majority of *GID1b-OE*-regulated genes showed similar AR-regulation in early Phase II (0h), whereas most of the genes differentially regulated with *GID1b-OE* showed the opposite regulation with after-ripening in late Phase II (12h) (Figs 7 and 8). This suggests that *GID1b* mainly contributes to dormancy loss in early Phase II–a crucial time for regulating the transition from quiescent to germinating seed. Some of the changes we are seeing in late Phase II with *GID1b-OE* may be negative feedback effects rather than the primary transcriptional changes resulting in dormancy loss. The accumulation of GID1b, GID1a, and GID1c proteins increased in response to after-ripening in Arabidopsis [46]. The nine genes showing similar transcriptional changes with *GID1b-OE* and after-ripening likely represent the subset of AR-regulated genes responding to increased GID1b protein levels with after-ripening (Figs 7 and 8).

If after-ripening and *GID1b-OE* rescue *sly1-2* seed germination by down-regulating DELLA through increased GID1-GA-DELLA complex formation, then we would expect *GID1b-OE*- and AR-regulated genes to be similarly regulated by GA and inversely by DELLA. While this held true for both *sly1-2* and L*er* AR-regulated genes, it did not always hold true for *GID1b-OE* particularly in late Phase II (DELLA-regulation defined by DvsWT or Cao et al. [49]) (Fig 8; [6]). Only 7 of the 22 *GID1b-OE* differentially-regulated genes were inversely regulated by DELLA (Figs 6, 7 and 8). A large portion of *GID1b-OE*-regulated genes at 12h were DELLA-regulated in the opposite direction as expected if *GID1b-OE*-regulation occurs through negative regulation of DELLA (Figs 6 and 8). It was also the case that a larger fraction of AR-regulated genes were inversely DELLA-regulated at the 12h than at the 0h timepoint (Figs 2C, 7 and 8; S1C Fig). Thus, more transcriptional effects from negative regulation of DELLA were apparent later in Phase II.

### There are likely many mechanisms that can alleviate seed dormancy

There are many genetic mechanisms contributing to seed dormancy and dormancy release. This study specifically defined DELLA/*sly1*-dependent and independent aspects of seed dormancy and dormancy release. Previous work also found that there were ABA-dependent and independent transcriptome changes associated with dormancy and after-ripening [8]. Of the genes regulated by after-ripening, 52% are DELLA-regulated and 79% are ABA-regulated (Fig 2B; [6,8]). Clearly, the DELLA- and ABA-regulated aspects of dormancy and after-ripening cannot be mutually exclusive. This is consistent with previous work showing that some GA-regulated genes are also ABA-regulated, and *vice versa* [14,42]. Moreover, DELLA directly regulates *XERICO*, a positive regulator of ABA biosynthesis [24]. Transcriptome studies in the highly dormant ecotype Cape Verde Islands have also defined transcriptome changes associated with both primary and secondary seed dormancy [5,7]. The real question is, what are the first proteins or transcripts to initiate the processes needed to germinate, and how is the behavior of these regulators altered by dormancy-breaking processes such as after-ripening? Future work may be able to identify these first events by examining either dry seeds or seeds earlier in imbibition (Phase I).

While some changes in gene expression as seeds transition from early to late Phase II may be involved in activating the germination program, others may prevent germination. There were genes whose transcript levels increased from 0h to 12h of imbibition solely in after-ripened seeds, solely in dormant seeds, and in both dormant and after-ripened *sly1-2* seeds (S7C Fig). The fact that there were a substantial number of genes down and up-regulated going from 0h to 12h of imbibition solely in DORMANT seeds, suggests that dormancy is not a passive process where seeds fail to induce genes needed for germination. Instead, it suggests that some genes may actively prevent germination of dormant seeds. Such genes may be preparing the seed to survive re-desiccation or may be inhibiting the activity of genes/proteins needed for germination. The Dekkers et al., [108] study made similar observations and defined dormancy-dependent genes and after-ripening-dependent genes in Cape Verde Islands in different tissues and across multiple imbibition timepoints.

## Materials and methods

### Plant materials and growth conditions

*Arabidopsis thaliana* ecotype Landsberg *erecta* (L*er*) wild-type, *sly1-2* and *sly1-2 GID1b-OE* in the L*er* background were previously described [34,41]. The *GID1b-OE* allele is an *HA*:*GID1b* translational fusion driven by the strong cauliflower mosaic virus 35S promoter in the *sly1-2* background. Arabidopsis used for all experiments were cultivated side-by-side in a Conviron^®^ growth chamber according to McGinnis et al. [35]. After harvest, seeds were stored in open tubes for 2 wk at room temperature and low humidity (≈15–30%) to allow dry after-ripening. Note that 2-wk-old seed means that seeds were stored under dry after-ripening conditions for 2 wk. Thereafter, seeds were stored in closed tubes at -20°C to preserve dormancy until plated. The long after-ripened *sly1-2*(AR) sample was a separate *sly1-2* batch grown in advance under the same conditions and stored at room temperature for 19 mo.

### Germination experiments

For the germination screen (Fig 1A), 80 to 100 seeds for each of the four seed batches were sterilized with 70% ethanol and 0.01% SDS for 5 minutes followed by 10% bleach and 0.01% SDS for a further 10 minutes, washed and plated on MS-agar plates containing 0.8% agar, 0.5× MS salts (Sigma-Aldrich), and 5 mM MES (2-(*N*-morpholino)ethanesulfonic acid), pH 5.5. The germination of the seed batches used for microarray analysis were scored daily for 8 d during incubation in the light at 22°C following cold stratification for 4 d at 4°C. Germination and testa rupture were also scored at the 12 h imbibition timepoint used for microarray analysis.

### Total RNA isolation from seeds

For RNA extraction, 20 mg of dry seed per sample was sown on filter paper moistened with sterile 0.5× MS salts buffered with 5mM MES, pH 5.5. Seeds were collected at two timepoints: 1) a 0h timepoint harvested immediately after cold stratification for 4 d at 4°C in the dark, and 2) a 12h timepoint incubated 12h in the light at 22°C following the 4 d cold stratification at 4°C. RNA isolation was performed using a phenol-chloroform extraction developed for tough tissues such as Arabidopsis seeds based on Oñate-Sánchez and Vicente-Carbajosa [109] with an additional chloroform extraction and the use of Phase Lock (5-PRIME) gel tubes to allow complete recovery of the aqueous phase without risk of organic phase contamination. RNA concentration was determined using a NanoDrop ND-2000c spectrophotometer (Thermo Scientific), and RNA quality checked by gel electrophoresis using RNA denatured at 70°C for 5 minutes in a formaldehyde dye mixture and run on a standard 1.1% agarose gel.

### Microarray and data analysis

Affymetrix ATH1 chip (22,810 genes) oligonucleotide-based DNA microarray analysis was performed using three biological replicates at the 0h and 12h imbibition timepoints, for each genotype: L*er* wt (2 wk old), dormant *sly1-2* (2 wk old), after-ripened *sly1-2* (19 mo old), and *sly1-2 GID1b-OE* (2 wk old). For each biological replicate, 2 μg of RNA was submitted to the Molecular Biology and Genomics Core Laboratory at Washington State University for synthesis of biotin-labeled cRNA, hybridization to ATH1 Arabidopsis microarray chips (Affymetrix), and chip scanning (http://crb.wsu.edu/core-laboratories/molecular-biology-and-genomics-core). Data analysis of raw CEL files was performed using the LIMMA package as part of the Bioconductor suite of tools in the R statistical programming environment [110–112]. Microarray raw data files are available at ArrayExpress (http://www.ebi.ac.uk/arrayexpress) under accession number E-MTAB-4782 [113]. Background correction and normalization was performed by Robust Multi-array Average (RMA), control probesets were removed, and significance was determined by False Discovery Rate (FDR) with α = 0.05 [114,115]. For genome-wide expression plots, the adjusted Fisher-Pearson standardized moment coefficient (G_1_) measurement of skew was calculated using the equation: *G*_*1*_ *= g*_*1*_ * sqrt(n(n-1)) / (n-2) [50].

Raw datasets from Cao et al. [49] and Carrera et al. [6] were obtained from ArrayExpress and NASCarrays (http://affymetrix.arabidopsis.info), respectively, and reanalyzed by the same methods to allow fair comparisons to the current study. In the Cao et al. [49] study, seeds were dry after-ripened for approximately 1 mo, then imbibed for 4 d at 4°C before harvesting for RNA isolation. In the Carrera et al. [6] study, the dormant L*er* were freshly harvested from yellow siliques, and the after-ripened L*er* were stored dry for 2 mo. Note that the freshly harvested L*er* were dormant, whereas the 2 week after-ripened L*er* wt seeds in Fig 1 were non-dormant and germinated efficiently.

Microarray analysis measures relative steady-state transcript levels, not active gene transcription. Thus, differential regulation could result from differences at the level of gene transcription or at the level of transcript stability. References to differential regulation or expression indicate differential regulation, but do not necessarily indicate differences in gene transcription. When referring to the differential regulation in A relative to B, or AvsB, up in AvsB means up-regulated in A (or down-regulated in B), whereas down in AvsB means down-regulated in A (or up-regulated in B).

In the GIDvsD comparison, very few differentially-regulated genes were identified considering the very different germination phenotypes. To ensure that the very high expression of *GID1b* was not causing artifactually low significance for other genes as a result of the multiple comparisons adjustment, significance testing was repeated with *GID1b* removed. This reanalysis of the data did not result in any change to the number of differentially-regulated genes in GIDvsD comparison at either imbibition timepoint. To determine whether differences in signal intensity might account for differences in the number of differentially-regulated genes, we also graphed signal intensities for *sly1-2* ARvsD and GIDvsD at both imbibition timepoints (S9 Fig). It was clear that the significant differences were observed over a wide range of signal intensities, allowing confidence that these differences were not artifacts due to low signal intensity.

### MicroarrayTools R package

R software tools were written for this study and are available through github in the microarrayTools package (https://github.com/bakuhatsu/microarrayTools). The microarrayTools package includes four functions: *getProbeID*, *venndia*, *TAGGITontology*, and *TAGGITplot*. *getProbeID* can be used to convert a gene or list of genes from AGI identifiers (e.g. At4g24210) or short gene names (e.g. SLY1) to ATH1 Affymetrix probe ids. Overlaps between comparisons were identified using the *venndia* function (Fig 2; S1 and S7 Figs). This function allows comparison of up- and down-regulated genes simultaneously and can be nested to simplify complex comparisons (overlaps of overlaps) (S1D Fig). In addition to plotting, overlapping genes can be returned as lists of up- and down-regulated genes for further analysis. For seed-specific gene ontology classification of differentially-regulated genes, an R pipeline was developed similar to the TAGGIT pipeline originally developed as an Excel macro by Carrera et al. [6]. The *TAGGITontology* function takes a list of significant genes and outputs a data frame for plotting and and an excel file containing hits. For searching gene descriptions *TAGGITontology* calls specialized functions written in C++ via the Rcpp package to greatly improve search speeds over pure R code [116]. The *TAGGITplot* function, which utilizes the ggplot2 package, automates plotting bar charts such as those published in Fig 4, S2 Fig and S3 Fig [117]. An example script is shown in S9 Fig.

### TAGGIT gene ontology analysis in R

Unlike standard Gene Ontology (GO), which identifies gene categories based on enrichment within a set of differentially regulated genes, TAGGIT quantifies enrichment for genes in a set of 26 specific categories defined for their involvement in seed dormancy and germination [6]. Genes were categorized a) by using the provided list of search terms for each category and comparing to the current gene descriptions, and b) by comparison to AGI identifiers from the original TAGGIT list (S2 Fig; S5 Table; [6]). Since gene annotation information, including descriptions, was pulled live from the ath1121501.db online database (Bioconductor) it was possible to identify TAGGIT genes that were not present in the original TAGGIT analysis [6,110]. This list of gene descriptions can be updated at any time by re-defining the myAnnot object. Using the *TAGGITontology* and *TAGGITplot* functions, it is possible to plot genes enriched for in the up-regulated fraction as red bars, and in the down-regulated fraction as blue bars. For simplicity, a higher degree of enrichment in the up-regulated fraction of a certain category was referred to as “more up-regulation” in this category, while a higher degree in the down-regulated fraction as “more down-regulation” in this category.

### Transcription factor target gene enrichment analysis

The web-based PlantGSEA toolkit (http://structuralbiology.cau.edu.cn/PlantGSEA/) with the Transcription Factor Targets (TFT) dataset identified known gene targets of transcription factors within differentially regulated genesets [51–53]. PlantGSEA identifies transcription factor targets based on published results of *in vivo* DNA-binding techniques such as ChIP coupled with DNA sequencing (ChIP-seq) and ChIP followed by microarray hybridization (ChIP-chip). The TFT dataset includes both targets of transcription factors and of transcriptional regulators that interact with a DNA-binding proteins, both referred to as TFs in this work. TFTs are considered “confirmed” if their function was proven by two or more approaches including *in vivo* evidence. Significantly enriched targets are presented in Table 1, the “All” category includes both “confirmed” and “unconfirmed” TFTs. Complete tables of TFT hits are available in S4 Table. Enrichment relative to the whole transcriptome was determined using a Fisher statistical test with the Yekutieli (FDR under dependency) correction for multiple testing adjustment with α = 0.06 [118,119].

### RT-qPCR analysis for microarray validation

RT-qPCR analysis for microarray validation was performed using gene-specific primers for select differentially regulated genes: *GASA2*, *GASA4*, *HSFA9*, *DOG1*, *XERICO*, and *SLY1* (Fig 9; S6 Fig). cDNA for RT-qPCR was synthesized from 1 μg of total RNA using the ProScript^®^ M-MuLV First Strand cDNA synthesis (New England Biolabs), and RT-qPCR analysis was conducted using the Lightcycler LightCycler^®^ FastStart DNA Master SYBR Green I kit (Roche). Primers were designed using the QuantPrime online tool (http://www.quantprime.de), with the exception of the previously published *HSFA9* and *DOG1* primers (S10 Fig; [120–122]). Reaction efficiencies were calculated based on dilution curves, all primer pair efficiencies were within 10% of each other and ±10% of 100% efficiency. Conditions for RT-qPCR were as follows: 10 minutes at 95°C (initial denature), followed by 45 cycles of 10 s at 95°C (denaturation), 5 s at the primer-specific annealing temperature (see S10 Fig), and 10 s at 72°C (extension). Changes in mRNA transcript abundance were quantified for the three biological replicates per gene using the Delta-Delta Ct method [123] and calculated relative to the constitutive *HOBBIT* (*HBT*; *At2g20000*) control [60]. The *HBT* control was selected based on the Graeber et al. [60] comparison of 12 Arabidopsis control genes in germinating seeds, where it was demonstrated to have the highest expression stability (lowest intersample transcript abundance variation) of the genes tested. Statistical testing was performed by pairwise Student’s t-test with Bonferroni-Holm correction for multiple comparisons with α = 0.07 (S6 Fig; [124]).

## Supporting information

S1 Fig Venn diagrams of differentially-regulated genesets to identify overlaps. (A) Overlap between genes that are DELLA/*sly1*-regulated at 0h (based on DvsWT) with those that are oppositely GA-regulated at 0h in Cao et al. [49] (0h DvsWT ∩ 0h *ga1-3* vs WT). (B) Overlap between genes differentially regulated with after-ripening of *sly1-2* at 12h with those of L*er* at 24 h (12h *sly1-2* ARvsD ∩ 24 h L*er* ARvsD). (C) Overlap between genes inversely regulated by the *sly1* mutation at 0h with genes differentially regulated with *sly1-2* after-ripening at 0h (0h WTvsD ∩ 0h *sly1-2* ARvsD). (D) Overlap of 12h *sly1-2* and 24 h L*er* after-ripening-regulated genes with those inversely regulated by the *sly1* mutation at 12h. The *sly1*-regulated genes are likely also DELLA-regulated. Throughout this study, up- (red) and down-regulated (blue) genes are based on FDR cutoff of p < 0.05.
(PDF)

S2 Fig Diagram of pipeline for TAGGIT gene ontology analysis with the *TAGGITontology* and *TAGGITplot* R functions. (PDF)

S3 Fig TAGGIT gene ontology analysis of GA- and DELLA-regulated transcriptome changes. Using reanalyzed datasets from Cao et al. [49] with timepoint equivalent to 0h in early Phase II (imbibed for 4 d at 4°C). (A) GA-regulated genes determined by the L*er* wt vs *ga1-3* comparison, (B) DELLA-regulated genes determined by the *ga1-3* vs *ga1-3 4x della* comparison, and (C) total GA-regulated and total DELLA-regulated genesets compared. The value on the x-axis shows the percentage of the total differentially regulated genes within a dataset.
(PDF)

S4 Fig Table with logFCs for differentially regulated genes with rescue of *sly1-2* germination by *GID1b*-overexpression. (PDF)

S5 Fig Plots of the correlation between RT-qPCR and microarray data for each comparison at 0h and 12h timepoints. (PDF)

S6 Fig Tables of p-values for pairwise comparisons of RT-qPCR data. Significant values are indicated in black text. Significance is based on pairwise t-tests with Bonferroni-Holm correction for multiple comparisons with α = 0.07.
(PDF)

S7 Fig Venn diagrams of differentially-regulated genesets to identify overlaps. (A) Overlap between genes differentially regulated with after-ripening of *sly1-2* at 12h with those that are GA-regulated based on the L*er* wt vs *ga1-3* comparison from Cao et al. [49] (12h *sly1-2* ARvsD ∩ 0h WT vs *ga1-3*). (B) Overlap between genes differentially regulated with after-ripening of L*er* wt at 24 h (Carrera et al. [6]) with those that are GA-regulated based on the L*er* wt vs *ga1-3* comparison (24 h L*er* ARvsD ∩ 0h WT vs *ga1-3*). (C) Overlap between genes differentially regulated in *sly1-2*(D) from 0h to 12h of imbibition with those of *sly1-2*(AR) from 0h to 12h of imbibition (*sly1-2*(AR) 12hvs0h ∩ *sly1-2*(D) 12hvs0h). Significance based on an FDR cutoff of p < 0.05.
(PDF)

S8 Fig Plots comparing intensities of microarray data after RMA normalization. Red circles indicate genes that were significantly differentially regulated (FDR, p < 0.05). Black circles were plotted with transparency of 25% such that darker areas indicate a larger accumulation of dots at the same location. Normalized probe intensities (log_2_ scale) were compared. A, 0h *sly1-2*(AR) (y-axis) versus 0h *sly1-2*(D) (x-axis). B, 12h *sly1-2*(AR) (y-axis) versus 12h *sly1-2*(D) (x-axis). C, 0h *sly1-2 GID1b-OE* (y-axis) versus 0h *sly1-2*(D) (x-axis). D, 12h *sly1-2 GID1b-OE* (y-axis) versus 12h *sly1-2*(D) (x-axis).
(PDF)

S9 Fig Example code for analyzing a dataset with *TAGGITontology* and plotting it with *TAGGITplot*. (PDF)

S10 Fig Table of primers used for RT-qPCR with primer sequences and annealing temperatures used in this study. (PDF)

S1 Table Complete tables of significant differences 1) in the DvsWT comparison, 2) with *sly1-2* after-ripening, and 3) with *GID1b*-overexpression, for both the 0h and 12h timepoints. (XLSX)

S2 Table Table of 1) DELLA-regulated genes that change with after-ripening, which likely involve DELLA-proteolysis, and 2) DELLA-regulated genes which likely do not involve DELLA-proteolysis. (XLSX)

S3 Table Table of translation associated genes identified by TAGGIT analysis. Tables represent enrichment in 1) the down-regulated fraction of 0h DvsWT, 2) the downregulated fraction of 12h DvsWT, and 3) up-regulated fraction of 24 h L*er* ARvsD (Carrera et al., [6]).
(XLSX)

S4 Table Complete table of PlantGSEA enrichment analysis. Transcription factor targets differentially regulated with *sly1-2* after-ripening at 0h and 12h.
(XLSX)

S5 Table TAGGITguideAGIs and TAGGITguideSearchTerms tables. Used by the TAGGITontology R function to classify genes into TAGGIT gene ontology categories based on lists of AGI locus identifiers, and lists of search terms corresponding to each category, respectively.
(XLSX)
