# Supplementary material for: Transcriptional mechanisms associated with seed dormancy and dormancy loss in the gibberellin-insensitive sly1-2 mutant of Arabidopsis thaliana
Source: PLoS One. 2017 Jun 19;12(6):e0179143. doi: 10.1371/journal.pone.0179143 (PMC5476249; doi:10.1371/journal.pone.0179143)
Supplement: S1 Fig — (A) Overlap between genes that are DELLA/sly1-regulated at 0h (based on DvsWT) with those that are oppositely GA-regulated at 0h in Cao et al. [49] (0h DvsWT ∩ 0h ga1-3 vs WT). (B) Overlap between genes differentially regulated with after-ripening of sly1-2 at 12h with those of Ler at 24 h (12h sly1-2 ARvsD ∩ 24 h Ler ARvsD). (C) Overlap between genes inversely regulated by the sly1 mutation at 0h with genes differentially regulated with sly1-2 after-ripening at 0h (0h WTvsD ∩ 0h sly1-2 ARvsD). (D) Overlap of 12h sly1-2 and 24 h Ler after-ripening-regulated genes with those inversely regulated by the sly1 mutation at 12h. The sly1-regulated genes are likely also DELLA-regulated. Throughout this study, up- (red) and down-regulated (blue) genes are based on FDR cutoff of p < 0.05. (PDF) [file pone.0179143.s001.pdf]

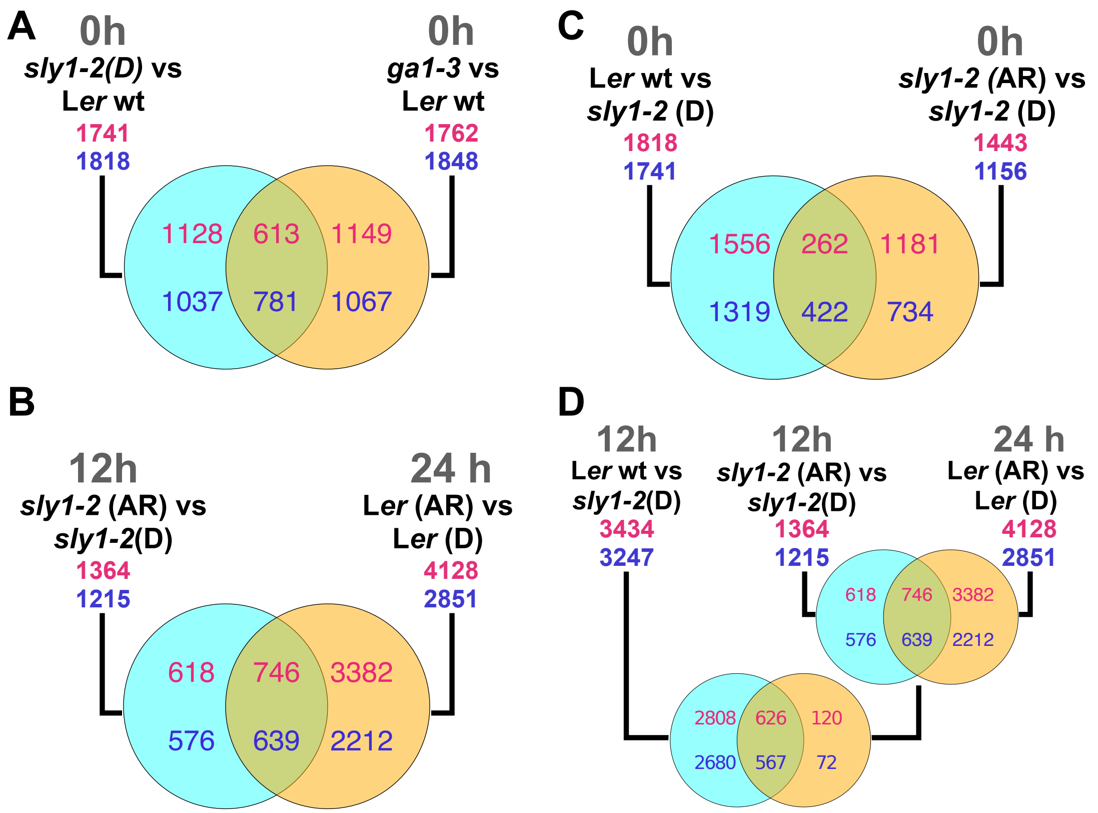

S1 Fig. Venn diagrams of differentially-regulated genesets to identify overlaps.

(A) Overlap between genes that are DELLA/*sly1*-regulated at 0h (based on DvsWT) with those that are oppositely GA-regulated at 0h in Cao et al. [49] (0h DvsWT ∩ 0h *ga1-3* vs WT). (B) Overlap between genes differentially regulated with after-ripening of *sly1-2* at 12h with those of L*er* at 24 h (12h *sly1-2* ARvsD ∩ 24 h L*er* ARvsD). (C) Overlap between genes inversely regulated by the *sly1* mutation at 0h with genes differentially regulated with *sly1-2* after-ripening at 0h (0h WTvsD ∩ 0h *sly1-2* ARvsD). (D) Overlap of 12h *sly1-2* and 24 h L*er* after-ripening-regulated genes with those inversely regulated by the *sly1* mutation at 12h. The *sly1*-regulated genes are likely also DELLA-regulated. Throughout this study, up- (red) and down-regulated (blue) genes are based on FDR cutoff of $p < 0.05$.
