# Supplementary material for: Transcriptional mechanisms associated with seed dormancy and dormancy loss in the gibberellin-insensitive sly1-2 mutant of Arabidopsis thaliana
Source: PLoS One. 2017 Jun 19;12(6):e0179143. doi: 10.1371/journal.pone.0179143 (PMC5476249; doi:10.1371/journal.pone.0179143)
Supplement: S2 Fig — (PDF) [file pone.0179143.s002.pdf]

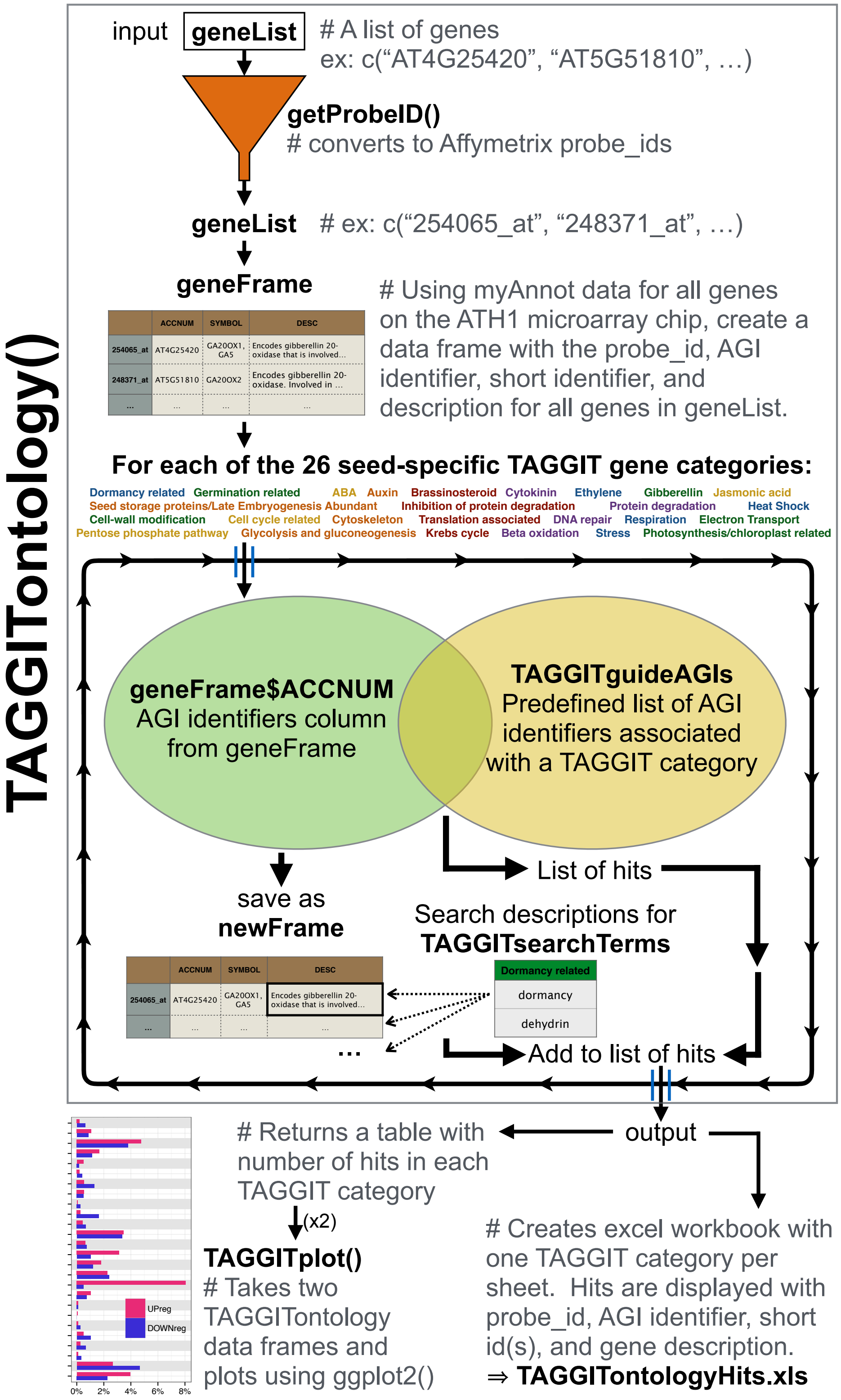

S2 Fig. Diagram of pipeline for TAGGIT gene ontology analysis with the *TAGGITontology* and *TAGGITplot* R functions.
