# Supplementary material for: Transcriptional mechanisms associated with seed dormancy and dormancy loss in the gibberellin-insensitive sly1-2 mutant of Arabidopsis thaliana
Source: PLoS One. 2017 Jun 19;12(6):e0179143. doi: 10.1371/journal.pone.0179143 (PMC5476249; doi:10.1371/journal.pone.0179143)
Supplement: S3 Fig — Using reanalyzed datasets from Cao et al. [49] with timepoint equivalent to 0h in early Phase II (imbibed for 4 d at 4°C). (A) GA-regulated genes determined by the Ler wt vs ga1-3 comparison, (B) DELLA-regulated genes determined by the ga1-3 vs ga1-3 4x della comparison, and (C) total GA-regulated and total DELLA-regulated genesets compared. The value on the x-axis shows the percentage of the total differentially regulated genes within a dataset. (PDF) [file pone.0179143.s003.pdf]

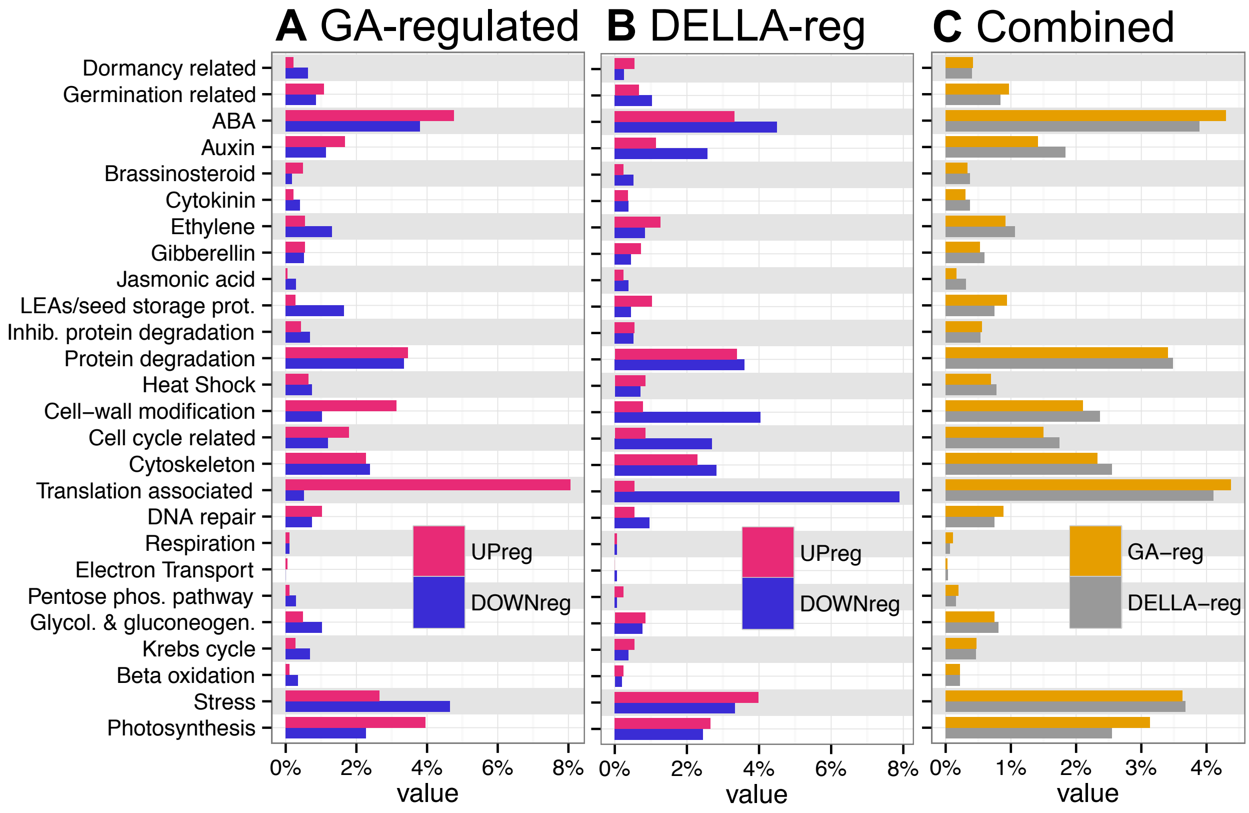

S3 Fig. TAGGIT gene ontology analysis of GA- and DELLA-regulated transcriptome changes.

Using reanalyzed datasets from Cao et al. [49] with timepoint equivalent to 0h in early Phase II (imbibed for 4 d at 4°C). (A) GA-regulated genes determined by the L*er* wt vs *ga1-3* comparison, (B) DELLA-regulated genes determined by the *ga1-3* vs *ga1-3 4x della* comparison, and (C) total GA-regulated and total DELLA-regulated genesets compared. The value on the x-axis shows the percentage of the total differentially regulated genes within a dataset.
