# Supplementary material for: Transcriptional mechanisms associated with seed dormancy and dormancy loss in the gibberellin-insensitive sly1-2 mutant of Arabidopsis thaliana
Source: PLoS One. 2017 Jun 19;12(6):e0179143. doi: 10.1371/journal.pone.0179143 (PMC5476249; doi:10.1371/journal.pone.0179143)
Supplement: S4 Fig — (PDF) [file pone.0179143.s004.pdf]

		GID1b-OE vs D			WT[a]	*ga1*[a] vs			*sly1-2*D vs WT		
AGI locus	Description	0h	12h	*GID1-OE*	vs *ga1*	*ga1 della*	GA	DELLA	0h	12h	*sly1*
At3g63010	GID1b	8.58	8.28	UP	-0.37	0.38	DOWN	UP	—	—	
At1g21630	EF hand family	2.77	2.95	UP	-0.44	0.41	DOWN	UP	—	—	
At5g46050	PTR3	1.26	—	UP	-1.82	2.15	DOWN	UP	—	0.60	UP
At5g54070	HSFA9	—	1.34	UP	—	—			1.85	2.75	UP
At4g09610	GASA2	—	1.29	UP	-0.94	—	DOWN		1.45	5.14	UP
At3g45970	EXPL1	—	1.01	UP	—	—			1.52	-1.79	UP/DOWN
At2g34740	PP2C protein	—	0.97	UP	—	—			1.75	1.50	UP
At3g22490	RAB28 LEA protein	—	0.91	UP	-2.95	2.82	DOWN	UP	1.34	4.63	UP
At5g45690	unknown protein	—	0.83	UP	-2.40	2.50	DOWN	UP	1.72	5.51	UP
At2g46240	BAG6	-2.69	-3.19	DOWN	—	—			—	—	
At2g46250	myosin-heavy chain rel.	-1.26	-3.39	DOWN	—	—			—	—	
At1g17430	α/β hydrolase fold	-1.10	—	DOWN	—	—			2.44	2.06	UP
At5g01740	NTF2 family	-1.07	—	DOWN	—	—			1.05	—	UP
At5g48850	SDI	-1.06	—	DOWN	—	—			-2.14	-0.70	DOWN
At5g58860	HORST	-1.00	—	DOWN	—	—			—	—	
At1g09200	histone H3.1	-0.93	—	DOWN	—	-2.17		DOWN	—	-1.68	DOWN
At1g22760	PAB3	-0.90	—	DOWN	—	—			0.62	—	UP
At5g56580	ANQ1, MKK6	-0.83	—	DOWN	0.70	—	UP		—	0.49	UP
At1g56190	phosphoglycerate kinase	-0.79	—	DOWN	—	—			1.10	0.60	UP
At5g07480	KUOX1	—	-1.28	DOWN	-2.04	1.95	DOWN	UP	1.89	4.26	UP
At2g44800	oxidoreductase	—	-1.13	DOWN	-2.42	2.37	DOWN	UP	—	2.91	UP
At2g40880	CYSa	—	-0.77	DOWN	—	—			0.66	-0.50	UP/DOWN
At2g16060	AHb1/GLB1	—	-0.73	DOWN	—	—			—	-2.47	DOWN

[a]Dataset from Cao *et al.*, 2006, equivalent to 0h timepoint

[b]*GID1b-OE*: *GID1b-OE*-regulated, GA: GA-regulated, DELLA: DELLA-regulated, *sly1*: *sly1*-regulated

S4 Fig. Table with logFCs for differentially regulated genes with rescue of *sly1-2* germination by *GID1b*-overexpression.
