# Supplementary material for: Transcriptional mechanisms associated with seed dormancy and dormancy loss in the gibberellin-insensitive sly1-2 mutant of Arabidopsis thaliana
Source: PLoS One. 2017 Jun 19;12(6):e0179143. doi: 10.1371/journal.pone.0179143 (PMC5476249; doi:10.1371/journal.pone.0179143)
Supplement: S6 Fig — Significant values are indicated in black text. Significance is based on pairwise t-tests with Bonferroni-Holm correction for multiple comparisons with α = 0.07. (PDF) [file pone.0179143.s006.pdf]

GASA2

		0h Ler (wt)	0h *sly1-2(D)*	0h *sly1-2(AR)*	0h *sly1-2 GID1b-OE*	12h Ler (wt)	12h *sly1-2(D)*	12h *sly1-2(AR)*	12h *sly1-2 GID1b-OE*
0h	Ler (wt)	—							
	sly1-2(D)	0.0001	—						
	sly1-2(AR)	0.0366	0.0401	—					
	sly1-2 GID1b-OE	0.0006	*0.7333*	*0.2821*	—				
12h	Ler (wt)	4.7E-10	2.7E-12	2.7E-11	5.8E-12	—			
	sly1-2(D)	0.0006	*0.7333*	*0.2821*	*0.9584*	5.8E-12	—		
	sly1-2(AR)	0.0366	4.3E-07	0.0001	2.2E-06	1.5E-08	2.1E-06	—	
	sly1-2 GID1b-OE	6.2E-06	*0.6070*	0.0023	*0.1227*	1.1E-12	*0.1227*	6.4E-08	—

GASA4

		0h Ler (wt)	0h *sly1-2(D)*	0h *sly1-2(AR)*	0h *sly1-2 GID1b-OE*	12h Ler (wt)	12h *sly1-2(D)*	12h *sly1-2(AR)*	12h *sly1-2 GID1b-OE*
0h	Ler (wt)	—							
	sly1-2(D)	3.2E-08	—						
	sly1-2(AR)	7.0E-06	0.0067	—					
	sly1-2 GID1b-OE	8.9E-08	*0.7979*	0.0364	—				
12h	Ler (wt)	6.8E-10	1.4E-13	1.4E-12	2.1E-13	—			
	sly1-2(D)	0.0001	0.0006	*0.7472*	0.0029	3.4E-12	—		
	sly1-2(AR)	0.0539	1.5E-09	1.2E-07	3.6E-09	1.2E-08	6.7E-07	—	
	sly1-2 GID1b-OE	1.3E-07	*0.7472*	0.0588	*0.7979*	2.6E-13	0.0058	5.1E-09	—

HSFA9

		0h Ler (wt)	0h *sly1-2(D)*	0h *sly1-2(AR)*	0h *sly1-2 GID1b-OE*	12h Ler (wt)	12h *sly1-2(D)*	12h *sly1-2(AR)*	12h *sly1-2 GID1b-OE*
0h	Ler (wt)	—							
	sly1-2(D)	0.0043	—						
	sly1-2(AR)	*0.8674*	0.0357	—					
	sly1-2 GID1b-OE	0.0025	*1.0000*	0.0194	—				
12h	Ler (wt)	1.5E-07	1.1E-09	4.1E-08	8.7E-10	—			
	sly1-2(D)	*0.8505*	0.0004	*0.1754*	0.0003	7.8E-07	—		
	sly1-2(AR)	0.0004	3.2E-07	0.0001	2.2E-07	0.0015	0.0035	—	
	sly1-2 GID1b-OE	*0.4926*	*0.0988*	*1.0000*	0.0636	2.0E-08	0.0636	1.8E-05	—

DOG1

		0h Ler (wt)	0h *sly1-2(D)*	0h *sly1-2(AR)*	0h *sly1-2 GID1b-OE*	12h Ler (wt)	12h *sly1-2(D)*	12h *sly1-2(AR)*	12h *sly1-2 GID1b-OE*
0h	Ler (wt)	—							
	sly1-2(D)	4.4E-07	—						
	sly1-2(AR)	1.9E-08	0.0546	—					
	sly1-2 GID1b-OE	4.0E-06	*0.2573*	0.0038	—				
12h	Ler (wt)	2.6E-11	7.0E-14	1.9E-14	1.6E-13	—			
	sly1-2(D)	1.9E-08	4.6E-12	8.7E-13	1.3E-11	1.8E-05	—		
	sly1-2(AR)	6.1E-07	3.1E-11	5.3E-12	1.1E-10	3.1E-07	0.0471	—	
	sly1-2 GID1b-OE	1.9E-08	4.6E-12	8.7E-13	1.3E-11	1.8E-05	*0.9482*	0.0471	—

XERICO

		0h Ler (wt)	0h *sly1-2(D)*	0h *sly1-2(AR)*	0h *sly1-2 GID1b-OE*	12h Ler (wt)	12h *sly1-2(D)*	12h *sly1-2(AR)*	12h *sly1-2 GID1b-OE*
0h	Ler (wt)	—							
	sly1-2(D)	0.0003	—						
	sly1-2(AR)	4.5E-06	*0.1839*	—					
	sly1-2 GID1b-OE	0.0005	*1.0000*	*0.1418*	—				
12h	Ler (wt)	*1.0000*	0.0021	2.2E-05	0.0031	—			
	sly1-2(D)	4.7E-07	0.0088	*0.8357*	0.0060	1.9E-06	—		
	sly1-2(AR)	0.0003	*1.0000*	*0.1904*	*1.0000*	0.0017	0.0107	—	
	sly1-2 GID1b-OE	4.2E-06	*0.1759*	*1.0000*	*0.1333*	2.0E-05	*0.8357*	*0.1865*	—

SLY1

		0h Ler (wt)	0h *sly1-2(D)*	0h *sly1-2(AR)*	0h *sly1-2 GID1b-OE*	12h Ler (wt)	12h *sly1-2(D)*	12h *sly1-2(AR)*	12h *sly1-2 GID1b-OE*
0h	Ler (wt)	—							
	sly1-2(D)	7.1E-07	—						
	sly1-2(AR)	0.0012	2.4E-09	—					
	sly1-2 GID1b-OE	3.4E-06	*1.0000*	7.4E-09	—				
12h	Ler (wt)	0.0423	1.4E-08	*0.6456*	4.7E-08	—			
	sly1-2(D)	3.4E-07	*1.0000*	1.4E-09	*0.6456*	7.6E-09	—		
	sly1-2(AR)	1.7E-05	3.0E-10	*0.2484*	7.9E-10	0.0075	1.9E-10	—	
	sly1-2 GID1b-OE	5.6E-07	*1.0000*	2.0E-09	*1.0000*	1.1E-08	*1.0000*	2.6E-10	—

S6 Fig. Tables of p-values for pairwise comparisons of RT-qPCR data.

Significant values are indicated in black text. Significance is based on pairwise t-tests with Bonferroni-Holm correction for multiple comparisons with α = 0.07.
