# Supplementary material for: Transcriptional mechanisms associated with seed dormancy and dormancy loss in the gibberellin-insensitive sly1-2 mutant of Arabidopsis thaliana
Source: PLoS One. 2017 Jun 19;12(6):e0179143. doi: 10.1371/journal.pone.0179143 (PMC5476249; doi:10.1371/journal.pone.0179143)
Supplement: S7 Fig — (A) Overlap between genes differentially regulated with after-ripening of sly1-2 at 12h with those that are GA-regulated based on the Ler wt vs ga1-3 comparison from Cao et al. [49] (12h sly1-2 ARvsD ∩ 0h WT vs ga1-3). (B) Overlap between genes differentially regulated with after-ripening of Ler wt at 24 h (Carrera et al. [6]) with those that are GA-regulated based on the Ler wt vs ga1-3 comparison (24 h Ler ARvsD ∩ 0h WT vs ga1-3). (C) Overlap between genes differentially regulated in sly1-2(D) from 0h to 12h of imbibition with those of sly1-2(AR) from 0h to 12h of imbibition (sly1-2(AR) 12hvs0h ∩ sly1-2(D) 12hvs0h). Significance based on an FDR cutoff of p < 0.05. (PDF) [file pone.0179143.s007.pdf]

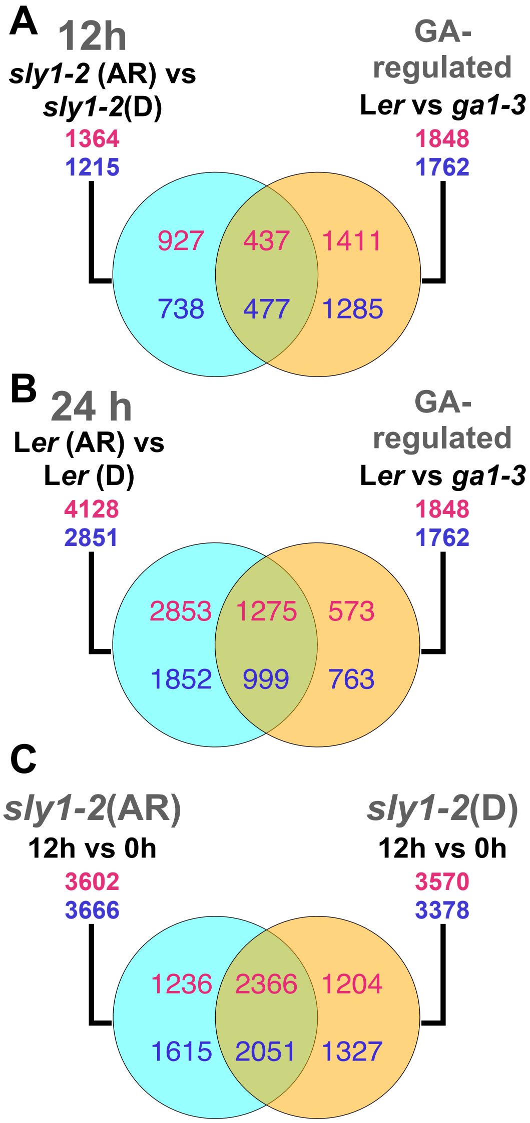


**S7 Fig. Venn diagrams of differentially-regulated genesets to identify overlaps.**

(A) Overlap between genes differentially regulated with after-ripening of *sly1-2* at 12h with those that are GA-regulated based on the L*er* wt vs *ga1-3* comparison from Cao et al. [49] (12h *sly1-2* ARvsD ∩ 0h WT vs *ga1-3*). (B) Overlap between genes differentially regulated with after-ripening of L*er* wt at 24 h (Carrera et al. [6]) with those that are GA-regulated based on the L*er* wt vs *ga1-3* comparison (24 h L*er* ARvsD ∩ 0h WT vs *ga1-3*). (C) Overlap between genes differentially regulated in *sly1-2*(D) from 0h to 12h of imbibition with those of

*sly1-2*(AR) from 0h to 12h of imbibition (*sly1-2*(AR) 12hvs0h ∩ *sly1-2*(D) 12hvs0h).

Significance based on an FDR cutoff of $p < 0.05$.
