# Supplementary material for: Transcriptional mechanisms associated with seed dormancy and dormancy loss in the gibberellin-insensitive sly1-2 mutant of Arabidopsis thaliana
Source: PLoS One. 2017 Jun 19;12(6):e0179143. doi: 10.1371/journal.pone.0179143 (PMC5476249; doi:10.1371/journal.pone.0179143)
Supplement: S8 Fig — Red circles indicate genes that were significantly differentially regulated (FDR, p < 0.05). Black circles were plotted with transparency of 25% such that darker areas indicate a larger accumulation of dots at the same location. Normalized probe intensities (log2 scale) were compared. A, 0h sly1-2(AR) (y-axis) versus 0h sly1-2(D) (x-axis). B, 12h sly1-2(AR) (y-axis) versus 12h sly1-2(D) (x-axis). C, 0h sly1-2 GID1b-OE (y-axis) versus 0h sly1-2(D) (x-axis). D, 12h sly1-2 GID1b-OE (y-axis) versus 12h sly1-2(D) (x-axis). (PDF) [file pone.0179143.s008.pdf]

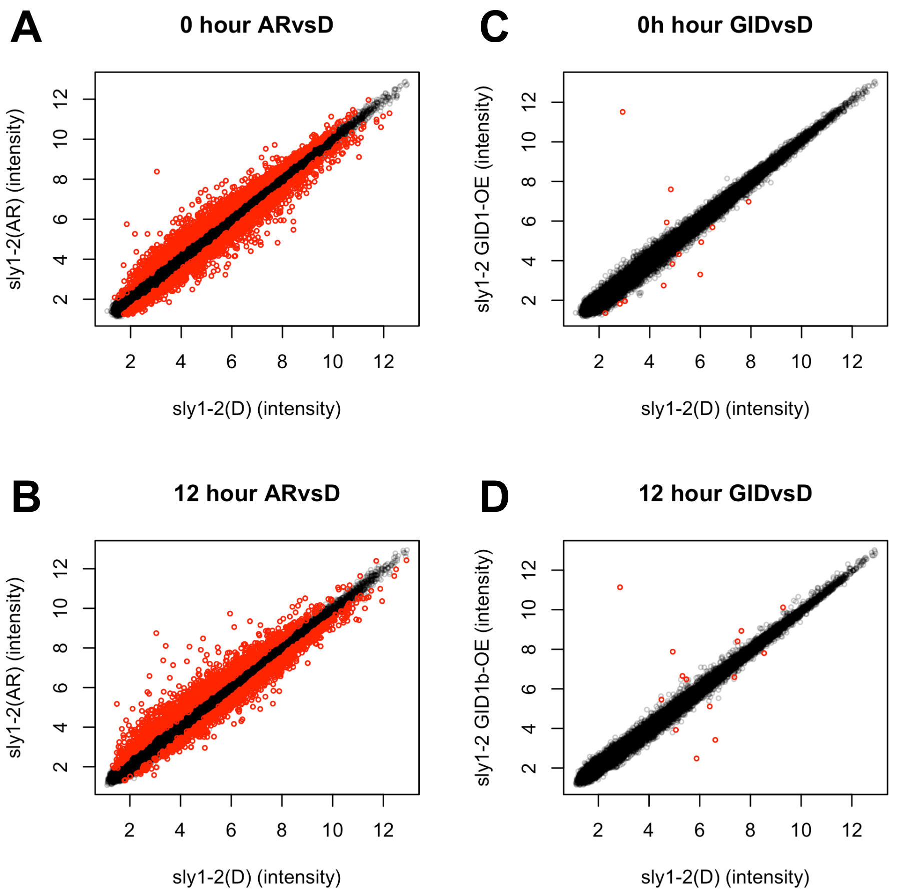

S8 Fig. Plots comparing intensities of microarray data after RMA normalization.

Red circles indicate genes that were significantly differentially regulated (FDR, $p < 0.05$). Black circles were plotted with transparency of 25% such that darker areas indicate a larger accumulation of dots at the same location. Normalized probe intensities (log_2 scale) were compared. A, 0h *sly1-2*(AR) (y-axis) versus 0h *sly1-2*(D) (x-axis). B, 12h *sly1-2*(AR) (y-axis) versus 12h *sly1-2*(D) (x-axis). C, 0h *sly1-2 GID1b-OE* (y-axis) versus 0h *sly1-2*(D) (x-axis). D, 12h *sly1-2 GID1b-OE* (y-axis) versus 12h *sly1-2*(D) (x-axis).
