# Supplementary material for: Transcriptional mechanisms associated with seed dormancy and dormancy loss in the gibberellin-insensitive sly1-2 mutant of Arabidopsis thaliana
Source: PLoS One. 2017 Jun 19;12(6):e0179143. doi: 10.1371/journal.pone.0179143 (PMC5476249; doi:10.1371/journal.pone.0179143)
Supplement: S9 Fig — (PDF) [file pone.0179143.s009.pdf]

```r
##################################################
## Author: Sven Nelson                          ##
## Example for running TAGGIT analysis          ##
## https://github.com/bakuhatsu/microarrayTools  ##
##################################################

#### Installing and loading the microarrayTools package: ####
## Install and load devtools for loading packages from GitHub
install.packages("devtools") # to allow us to install packages from GitHub
library(devtools)
# microarrayTools also relies on the ath1121501.db package from Bioconductor.
# Bioconductor packages cannot be automatically installed like other R dependencies.
# try http:// if https:// URLs are not supported
source("https://bioconductor.org/biocLite.R")
## If you haven't previously installed Bioconductor, run the next line.
biocLite() # Installs Bioconductor base packages, takes a long time for a fresh install.
## To install the ath1121501.db package
biocLite("ath1121501.db")
## Install microarrayTools functions: TAGGITontology, TAGGITplot, getProbeID, and venndia
install_github("bakuhatsu/microarrayTools") # installing from GitHub: username/library
library(microarrayTools) # To load the package

#### Setting up lists of up-/down-regulated genes for TAGGITontology(): ####
## Create a vector of probe_ids (ex 248961_at) or AGI identifiers (ex AT5G45650)
# NOTE: "etc..." is used to indicate that the list continues, do not use in real code.
GeneSet_UP <- c("AT4G25420", "AT5G51810", "AT5G07200", "AT1G30040", etc...) # UP-reg
GeneSet_DOWN <- c("AT4G18350", "AT4G19170", "AT3G24220", "AT1G78390", etc...) # DOWN-reg
# If you have loaded microarray data into R, you can easily create a list from topTable
# Please see the Bioconductor documentation for instructions for use of topTable
# NOTE: Replace "YourData.rma" and "ComparisonCoef" with appropriate input from your data
GeneSet_UP <- row.names(subset(topTable(YourData.rma, coef=ComprisonCoef, adjust="fdr",
                        sort.by="B", number=Inf, p.value = 0.05)), logFC>0) # only up-regulated genes
GeneSet_DOWN <- row.names(subset(topTable(YourData.rma, coef=ComprisonCoef, adjust="fdr",
                        sort.by="B", number=Inf, p.value = 0.05)), logFC<0) # only down-regulated genes

#### Test run using the provided datasets in GeneSetdata: ####
# Example data is provided, use the following code to load it
data("GeneSetdata") # loads GeneSet_UP and GeneSet_DOWN example data.
## Make a list containing two vectors: 1) up-regulated genes, and 2) down-regulated genes
GeneSet <- list()
GeneSet$UP <- GeneSet_UP # A vector of upregulated genes
GeneSet$DN <- GeneSet_DOWN # A vector of down-regulated genes
## Create TAGGITontology objects (may take a few min, but has built-in progress bar)
# Returns dataframe for plotting and outputs an excel sheet of hits to the working dir.
GeneSet_TAGGIT_UP <- TAGGITontology(GeneSet$UP, outputFileName = "TAGGIThits_UP.xlsx")
GeneSet_TAGGIT_DN <- TAGGITontology(GeneSet$DN, outputFileName = "TAGGIThits_DN.xlsx")
## Plot the results of the TAGGIT analysis using ggplot2 via the TAGGITplot function
# Comparing UP and DOWN regulated genesets
TAGGITplot(GeneSet$UP, GeneSet$DN, GeneSet_TAGGIT_UP, GeneSet_TAGGIT_DN, title = "")
## Export the image: for best results export in EPS (vector) format
## For output like Figure 4 in the publication, export at 400x511 resolution

#### Total differentially regulated genes (up and down combined) for two comparisons: ####
## Create two lists as before: GeneSet_AvsB and GeneSet_CvsD
## Create TAGGITontology objects (example genesets not provided)
GeneSet_TAGGIT_AvsB <- TAGGITontology(GeneSet_AvsB)
GeneSet_TAGGIT_CvsD <- TAGGITontology(GeneSet_CvsD)
## Plot the results of the TAGGIT analysis using ggplot2 via the TAGGITplot function
TAGGITplot(GeneSet_AvsB, GeneSet_CvsD, GeneSet_TAGGIT_AvsB, GeneSet_TAGGIT_CvsD, A = "AvsB", B = "CvsD", title = "")
## Export the image: for best results export in EPS (vector) format
## For output like Supplemental Fig. S3, export at 400x511 resolution
```

S9 Fig. Example code for analyzing a dataset with *TAGGITontology* and plotting it with *TAGGITplot*.
