# Supplementary material for: Transcriptional mechanisms associated with seed dormancy and dormancy loss in the gibberellin-insensitive sly1-2 mutant of Arabidopsis thaliana
Source: PLoS One. 2017 Jun 19;12(6):e0179143. doi: 10.1371/journal.pone.0179143 (PMC5476249; doi:10.1371/journal.pone.0179143)
Supplement: S10 Fig — (PDF) [file pone.0179143.s010.pdf]

AGI locus	Gene	T_a	Direction	Name	Primer sequence (5'-3')	Reference
At4g09610	*GASA2*	64°C	forward	GASA2-qRT1-F	TGATGGTGCAAAGGTCGGTGAAG	current study
			reverse	GASA2-qRT1-R	TCTTTGCATCTCCCACCACAATCG	
At5g15230	*GASA4*	68°C	forward	GASA4-qRT1-F	ATGTGAAGTGGAGCCAGAAACG	current study
			reverse	GASA4-qRT1-R	ATTCCGATGGGCATTGGGTACG	
At5g54070	*HSFA9*	68°C	forward	HSFA9-qRT1-F	AGACGGCAACGGAGACCGTCACCGTTGAAAGAG	Guan et al., 2013
			reverse	HSFA9-qRT1-R	TTGGGAAGTAGATTCTCTGAGAACTCGTAAGAATCC	
At5g45830	*DOG1*	52°C	forward	DOG1-qRT1-F	ATGGGATCTTCATCAAAGAAC	Mortensen et al., 2011
			reverse	DOG1-qRT1-R	CTTACGAAGCTTGTTATCATTATC	
At2g04240	*XERICO*	70°C	forward	XERICO-qRT2-F	ACCTTGAGGAGTTCAGGAACCG	current study
			reverse	XERICO-qRT2-R	GTTTCTTGCACCTGCACAAGCTC	
At4g24210	*SLY1/sly1-2*	66°C	forward	SLY1-qRT3-F	TCTGTTGTGTTGGCGCTTGGTG	current study
			reverse	SLY1-qRT2-R	AGAGGCCAGAGGTAAAGAGAGTGG	
At2g20000	*HOBBIT*	65°C	forward	HBT-qRT2-F	TCGCTTAGCTCCTCAATCTTGGTG	current study
			reverse	HBT-qRT2-R	TCTTCAGTGCGGTCTCATGGTC	

S10 Fig. Table of primers used for RT-qPCR with primer sequences and annealing temperatures used in this study.
